# Effectiveness of Mobile Apps to Promote Health and Manage Disease: Systematic Review and Meta-analysis of Randomized Controlled Trials

**DOI:** 10.2196/21563

**Published:** 2021-01-11

**Authors:** Sarah J Iribarren, Tokunbo O Akande, Kendra J Kamp, Dwight Barry, Yazan G Kader, Elizabeth Suelzer

**Affiliations:** 1 Department of Biobehavioral Nursing and Health Informatics University of Washington Seattle, WA United States; 2 Department of Pediatrics Sanford Health Bemidji, MN United States; 3 Division of Gastroenterology School of Medicine University of Washington Seattle, WA United States; 4 Enterprise Analytics Seattle Children’s Hospital Seattle, WA United States; 5 Medical College of Wisconsin Libraries Medical College of Wisconsin Milwaukee, WI United States

**Keywords:** systematic review, mobile apps, mobile phone

## Abstract

**Background:**

Interventions aimed at modifying behavior for promoting health and disease management are traditionally resource intensive and difficult to scale. Mobile health apps are being used for these purposes; however, their effects on health outcomes have been mixed.

**Objective:**

This study aims to summarize the evidence of rigorously evaluated health-related apps on health outcomes and explore the effects of features present in studies that reported a statistically significant difference in health outcomes.

**Methods:**

A literature search was conducted in 7 databases (MEDLINE, Scopus, PsycINFO, CINAHL, Global Index Medicus, Cochrane Central Register of Controlled Trials, and Cochrane Database of Systematic Reviews). A total of 5 reviewers independently screened and extracted the study characteristics. We used a random-effects model to calculate the pooled effect size estimates for meta-analysis. Sensitivity analysis was conducted based on follow-up time, stand-alone app interventions, level of personalization, and pilot studies. Logistic regression was used to examine the structure of app features.

**Results:**

From the database searches, 8230 records were initially identified. Of these, 172 met the inclusion criteria. Studies were predominantly conducted in high-income countries (164/172, 94.3%). The majority had follow-up periods of 6 months or less (143/172, 83.1%). Over half of the interventions were delivered by a stand-alone app (106/172, 61.6%). Static/one-size-fits-all (97/172, 56.4%) was the most common level of personalization. Intervention frequency was daily or more frequent for the majority of the studies (123/172, 71.5%). A total of 156 studies involving 21,422 participants reported continuous health outcome data. The use of an app to modify behavior (either as a stand-alone or as part of a larger intervention) confers a slight/weak advantage over standard care in health interventions (standardized mean difference=0.38 [95% CI 0.31-0.45]; I2=80%), although heterogeneity was high.

**Conclusions:**

The evidence in the literature demonstrates a steady increase in the rigorous evaluation of apps aimed at modifying behavior to promote health and manage disease. Although the literature is growing, the evidence that apps can improve health outcomes is weak. This finding may reflect the need for improved methodological and evaluative approaches to the development and assessment of health care improvement apps.

**Trial Registration:**

PROSPERO International Prospective Register of Systematic Reviews CRD42018106868; https://www.crd.york.ac.uk/prospero/display_record.php?RecordID=106868

## Introduction

### Background

The health care field is experiencing exponential growth in the use of mobile apps to deliver interventions aimed at modifying behavior to promote health and manage disease. Behavior change interventions are broadly defined as “coordinated sets of activities designed to change specific behavior patterns” [1]. Traditionally, behavior change interventions are resource intensive and difficult to scale [2]. The enthusiasm for apps stems from their broad reach and capacity to perform multiple functions, including sophisticated features that can enhance person-centered care and improve health outcomes [3]. Research into the effectiveness of health apps to change behaviors is in its early stage, and there is no clear consensus on which specific features of apps can assist in behavior change [4]. Furthermore, most apps contain only a few features that could be considered to have the potential to change behavior [5]. Examples of features within apps that may promote health behavior change include *reminders or notifications* (eg, to prompt patients to take their medication at a specified time), *tracking* activity (eg, to encourage increased physical activity), goal planning, and tailored *information* (eg, provide information on the consequences of continuing a behavior) [6]. Despite the field being in its infancy and the lack of consensus on the efficacy of apps to promote health and manage disease, the use of health apps has become increasingly common [7].

With an estimated 325,000 health care–related apps now in existence [8], the global health app market is expected to reach US $236 billion by 2026 [9]. However, systematic reviews assessing the effectiveness of mobile apps for the management of various conditions such as asthma [10], cardiovascular disease [11], diabetes [12], physical and mental health [13], self-management of medication [14], and smoking cessation [15] have shown mixed results [16].

Similarly, findings from studies exploring the impact of app features on health outcomes have been mixed. For instance, Bonoto et al [12] observed that when the number of features in an app was more than 2, the efficiency of the app appeared to increase. However, other researchers have reported inconclusive results regarding the number of features [17,18]. To inform the future direction of health app development, there is a need to understand if certain features or *active ingredients*, such as self-tracking, feedback, and journaling, within apps are important contributors to improved outcomes [1]. Thus far, only a few systematic reviews of rigorously evaluated apps (eg, through randomized controlled trials [RCTs]) have been performed to assess which features are found in successful apps. In addition, these reviews have typically only looked at disease-specific apps [12,15,18-21]. Furthermore, none of those studies specifically evaluated which features of the apps had an effect on the outcomes.

### Objectives

Therefore, a broader review of this technology is warranted, given the widespread availability of health apps and the current gap in knowledge in the understanding of their effectiveness on health outcomes or the features that may have an effect on the outcomes. This study aims to summarize the evidence from RCTs of the effect of health apps on health outcomes. Our secondary aim is to explore the effect of features present in studies that reported a statistically significant difference in health outcomes (eg, feature count and which features were more likely to have a positive effect on the outcome). To this end, all studies rigorously evaluating a health app published between 2008 and early 2019 were reviewed and analyzed.

## Methods

This study was designed and reported in accordance with the PRISMA (Preferred Reporting Items for Systematic Reviews and Meta-Analysis) statement [22]. A protocol outlining the methods of this systematic review was registered in the International Prospective Register of Systematic Reviews (PROSPERO, CRD42018106868).

### Search Strategy

The search strategy was developed and executed in consultation with an experienced research librarian (ES). We executed our original search on October 2016 and completed an updated search on March 2019. The search strategy was created for Ovid MEDLINE using a combination of MeSH (Medical Subject Heading) terms, keywords, and phrases (Multimedia Appendix 1). The search terms targeted mobile apps, a broad range of disease/illnesses, and health-related outcomes. The strategy was translated for other databases—Scopus, PsycINFO, CINAHL, Global Index Medicus, Cochrane Central Register of Controlled Trials, and the Cochrane Database of Systematic Reviews—using their respective thesaurus terms and advanced search features. A manual search using reference lists of retrieved citations was conducted for other relevant studies.

### Eligibility Criteria

Textbox 1 summarizes the PICOS (participants, interventions, comparison, outcomes, and study design) to define the inclusion criteria strategy. A behavior change intervention is broadly defined as a “coordinated set of activities designed to change a specified behavior pattern” [2]. Given this broad definition, we included studies in which an intervention used a mobile health (mHealth) app to change a behavior to promote health or manage disease, whether or not authors explicitly labeled the intervention as *behavior change technique*. For example, apps that included techniques to increase exercise, improve medication adherence, or self-manage a chronic disease were included. Multimedia Appendix 2 [1] describes and defines the app features. There is currently no consensus on all app features and their definitions. We first developed this list of features based on the literature [20,23] and then, by consensus within the team, iteratively expanded and refined from our review of the included studies. We then assessed how the features corresponded to behavior change techniques [24]. We excluded apps that only collected data passively, with no other intervention or behavior change component (eg, step count collection, blood glucose automatic readings/continuous glucose reading). As app stores from which users could download apps were first launched in 2008, we used this year for the start of search [25]. We excluded articles published in languages other than English or Spanish, not meeting rigorous evaluation criteria, and reporting on behavior change interventions that did not include an app for delivery. In addition, the following studies were excluded: studies on app-based interventions targeting health care providers (eg, training, evaluation of prescription habits, diagnostic assistance, medical information references); interventions designed for nonmobile devices (eg, web-based interventions for computer use), using only wearable devices, or if the intervention was delivered within a health care facility (eg, hospital unit); testing of a smart-health device (eg, blood pressure monitoring, environmental sensors, blood glucose monitoring) or for monitoring a device (eg, pacemaker); reporting on app development; nonrigorously designed studies (nonrandomized studies, not controlled, quasi-experimental); virtual reality studies; systematic review of mobile apps; assessment of web-based networking (eg, blogs, Facebook groups); devices used in health care settings (eg, tablet-based intervention in a hospital setting); use of drones; and app only for notifying users of test results.

**Participants, interventions, comparison, outcomes, and study design criteria.**

Inclusion and exclusion criteria

Population

- Adults or children with any disease or health-related issue.

Intervention

- Any intervention using a mobile health app aimed to modify a behavior to promote health and manage disease whether or not authors explicitly labeled the intervention as “behavior change technique.” For example, an intervention that allows users to input data, receive feedback, connect with health professionals, learn about a disease, or manage their illness or disease.
- Intervention could have any length of follow-up on outcomes.

Comparator

- Routine practice
- Usual care
- Control
- Attention control

Outcomes

- Any direct health outcome that could be assessed for clinical effectiveness, such as medication adherence, treatment outcome (eg, blood pressure, hemoglobin A_1c_), health care promotion (eg, cardiovascular disease screening), behavior change (eg, smoking cessation, weight loss), scores measured using any validated standard instrument (eg, pain level, Patient Health Questionnaire for depression, quality of life).

Setting

- Participants in any country in their natural environment (eg, home, community) and not in a controlled health care setting (eg, hospital).

Study design

- Rigorous evaluation conditions: parallel randomized controlled trials (RCTs), cluster RCTs, quasi RCTs, controlled before-after studies, or interrupted time series studies with at least three time points before and after the intervention.

### Study Screening and Selection

Studies were screened for eligibility in duplicates under blinded conditions by 2 independent reviewers (SI, KK, YK, Hannah Erdy, and TA) as the best practice for systematic reviews [22]. Covidence software was used to aid in this process. Search results were first screened by title and abstract, and any studies that appeared to meet the eligibility criteria or where eligibility was unclear progressed to full-text screening. Next, 2 independent reviewers screened the full texts to determine eligibility for inclusion in the review. Results from each round of screening were compared and discussed until consensus was attained. When more than one publication referred to the same trial, the publication reporting on the primary outcomes of the study was selected. Reports of ongoing trials were excluded.

### Risk of Bias

The quality of the studies was assessed using the Cochrane Risk of Bias tool for RCTs [26]. We assessed each study for random sequence generation, allocation concealment, blinding of participants, blinding of personnel, blinding of outcome assessment, incomplete outcome data, selective reporting, and other sources of bias as low, unclear, or high. If intention-to-treat analysis was conducted, the study was considered to have a low risk for completeness of outcome data.

### Data Extraction and Variable Definitions

A standardized data extraction form was developed to include standard data (eg, title, year published, health condition) and study-specific data (eg, app features, level of personalization). Data extracted from each full-text paper included the following: title, publication year, authors, country, intervention name, health condition addressed, participant population age, study design, intervention focus, intervention description, mode of intervention delivery (eg, stand-alone app or app as part of a larger intervention such as an in-person component), comparator, intervention frequency, follow-up time, sample size calculation, use of theoretical framework to guide and develop intervention, app name, app features, level of personalization, health outcome, sample size at randomization and at final analysis, and outcome results. Features were extracted as reported or not reported. In cases where the features were not explicitly listed, we reviewed supplementary files, screenshots, or any source cited by the authors (protocol; website; or previous publications by authors detailing intervention development, testing, or initial evaluation) to determine if features were present or not. The level of personalization of the app was categorized as one-size-fits-all, static (one-time tailoring to individual), or dynamic (adaption occurs periodically or in real time during intervention). For health outcomes, we extracted the primary health outcome used to power the study; if there were multiple outcomes or if the power calculation was not reported, we selected the main health outcome. If the health outcome was a secondary outcome, this was noted. Where studies compared more than 2 interventions, we extracted the data from the control and the intervention groups that represented the most stand-alone app. Outcome data were extracted from the longest (last) follow-up. We abstracted summary measures such as means, SDs, event counts, and total n, calculating SD values from other summary metrics (eg, SE, CIs) when the raw value was not included in a study [27,28]. These data were compiled in a spreadsheet independently by 2 of the authors for a subset of 20 articles and were reviewed by the team for consensus of data extraction. Data extraction was then continued by one author and verified by a second extractor. When there was uncertainty for any data point, the article was reviewed as a team, and disagreements were resolved by discussion.

### Narrative Synthesis for Feature Definition and Outcome Categorization

Initial narrative synthesis is recommended before undertaking a quantitative synthesis of complex interventions to look at patterns and characteristics of the data identified [27]. Recommendations include organizing studies into logical categories possibly related to design, outcome, or intervention type [29]. Therefore, we used a thematic analysis to develop intervention outcome behavior change categories. These categories included nutrition and physical activity (eg, weight loss), mental health management (eg, reduction of depressive symptoms), medication adherence, general health and well-being (eg, quality of life), diabetes management (eg, hemoglobin A_1c_ management), management of other chronic diseases (eg, lowered blood pressure), and cessation/harm reduction (eg, days no drug use, reduced alcohol consumption, smoking cessation). Country income levels (eg, high and low income) were classified according to the World Bank listing [30].

### Statistical Analysis

#### Meta-analysis

We used a random-effects model to calculate pooled effect size estimations [31], where study heterogeneity was assessed using I^2^ and the Sidik-Jonkman estimator for calculating τ^2^ and prediction intervals [32]. Random effects analysis was chosen over a fixed-effect model because by calculating both within- and between-study variance (τ^2^), the relative weights assigned to each study are more balanced under the random-effects model than they are under a fixed-effect model, which only considers n for each study. Standardized mean differences (SMDs) were calculated as Hedges *g* for continuous measures and odds ratio for binary measures; when extracted data did not contain SDs but contained other statistical information (*t* values, *P* values, and CIs), we used those variables to calculate effect sizes following the formulae in the Cochrane Handbook [33]. Funnel plots were used to assess publication bias.

#### Sensitivity Analysis

Sensitivity analysis was conducted by performing subgroup analysis based on follow-up time, stand-alone app interventions, level of personalization, and pilot studies.

#### App Feature Analysis

We used logistic regression to examine the effect of app features detailed in Multimedia Appendix 2. We chose an outcome of an absolute SMD of 0.5 or greater as indicative of a successful app, which was modeled as the outcome variable in the logistic regression.

Statistical analyses were performed using R 3.6.2 (R Core Team 2019).

## Results

### Study Selection

**Figure 1:**
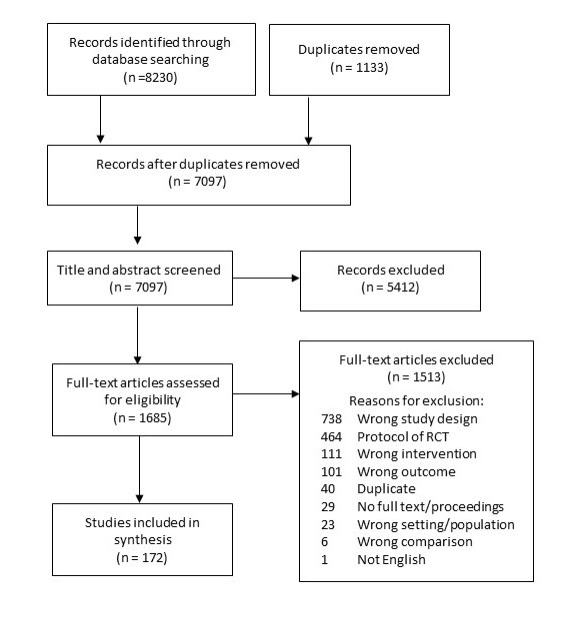
PRISMA (Preferred Reporting Items for Systematic Reviews and Meta-Analyses) flow diagram. RCT: randomized controlled trial.

From the database searches, 8230 records were initially identified. Of these, 172 met the inclusion criteria and were included in this review. See Figure 1 for the PRISMA flowchart of the study selection, including the rationale for the exclusion of full-text articles.

### Study Characteristics

**Figure 2:**
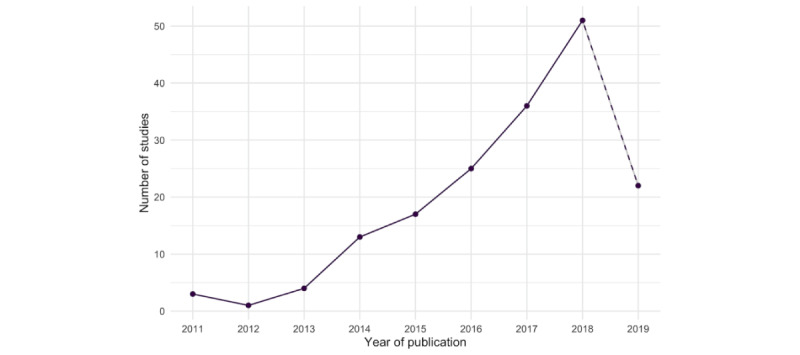
Publication of included studies per year.

Key study characteristics are summarized in Table 1. Most studies were parallel design or cluster RCTs (169/172, 98.3%), 2 were randomized crossover, and 1 was a randomized factorial design. A total of 34.3% (59/172) of the studies were characterized as pilot RCTs. Studies were predominantly conducted in high-income countries (164/172, 94.3%), with the United States having the highest count (69/172, 46.6%). Only 1 study was conducted in a low-income country (Ghana). The number of publications per year has steadily increased since 2012 (Figure 2).

**Table 1:** Study characteristics (N=172), n (%).

Characteristic		Value
**Reported as a randomized pilot study**		
	No	115 (66.1)
	Yes	59 (33.9)
**Region/country (number of studies per country)**		
	North America (USA-69, Canada-6, Mexico-1)	76 (43.7)
	Europe (UK-8, Spain, Sweden, Denmark, Italy, Netherlands)	49 (28.2)
	Asia (Korea-8, China-8, Japan-3, Taiwan-2)	26 (14.9)
	Oceania (Australia-16, New Zealand-3)	19 (10.9)
	Middle East, North Africa, Greater Arabia (Israel-2)	2 (1.2)
	Sub-Saharan Africa (Ghana)	1 (0.6)
	Multi-country	1 (0.6)
**Participant condition**		
	Chronic disease (Diabetes-28, Cardiovascular Disease-13, Pulmonary disease-9, HIV-4)	55 (33.2)
	Mental health (eg, depression, anxiety)	48 (27.8)
	Overweight/obese/physical inactivity/diet	47 (27.2)
	Substance use/abuse (eg, alcohol, nicotine, drugs)	10 (5.8)
	Cancer	7 (4.1)
	Neurological/musculoskeletal (eg, back pain, arthritis)	7 (4.1)
**Behavior assessed (health outcome)**		
	Nutrition and physical activity (eg, weight loss)	61 (34.7)
	Mental health management (eg, reduce depressive symptoms)	31 (17.9)
	Diabetes management (eg, hemoglobin A_1c_ reduction)	22 (12.7)
	General health/well-being (eg, quality of life, sleep quality)	20 (11.7)
	Medication adherence	15 (8.7)
	Cessation/harm reduction (eg, days no drug/alcohol use, smoking cessation)	13 (7.5)
	Management of other chronic diseases (eg, reduce blood pressure)	12 (6.9)
**Participant age category**		
	Adult	145 (83.2)
	Mix of age groups	18 (10.3)
	Adolescent	6 (3.5)
	Pediatric	3 (1.7)
	Older adult	2 (1.2)
**Comparator group**		
	Usual/standard of care	102 (58.6)
	Attention control	45 (25.9)
	Waitlist control	27 (15.5)
**App developed for research or clinically/commercially available**		
	Research	87 (50.5)
	Commercial/clinical	77 (44.3)
	Unclear	10 (5.7)
**App intervention type**		
	Stand-alone intervention	108 (62.1)
	Part of a larger intervention	66 (37.9)
	App+in person	22 (12.6)
	App+wearable	19 (10.9)
	App+2 other (eg, phone call+wearable, in person+patient portal)	15 (8.6)
	App+1 other (eg, support specialist, phone call, text messaging)	10 (5.7)
**Intervention frequency**		
	Daily/as needed	125 (71.8)
	Weekly	18 (10.3)
	Unclear	25 (14.4)
	Other	6 (3.4)
**Follow-up time (extracted last follow-up time)**		
	<1 month	10 (5.7)
	1-6 months	135 (77.6)
	7-12 months	25 (13.8)
	>12 months	4 (2.3)
**Reported use of behavior change theory for app development**		
	Yes	68 (39.1)
	No/not clearly reported	106 (60.9)
**Reported a power analysis**		
	Yes	103 (59.2)
	No/not clearly reported	71 (40.8)
**Reported intention-to-treat analysis**		
	Yes	96 (55.2)
	No/not clearly reported	78 (44.8)
**Level of personalization**		
	One-size-fits-all	74 (43.0)
	Static (one-time tailoring to individual)	23 (13.4)
	Dynamic (adaption occurs periodically or in real time during intervention)	75 (43.6)

### Participants

Sample sizes ranged from 6 to 14,228, with a total of 53,331 participants across the 172 studies. Overall, 83.1% (143/172) of studies targeted adults, whereas 8.1% (14/172) targeted exclusively pediatric patients or adolescents, and 0.6% (1/172) of the studies targeted older adults. The most common conditions of the participants were chronic diseases (55/172, 31.9%), mental health disorders (48/172, 27.9%), and overweight or obesity (45/172, 26.2%).

### Methodology

Control groups either received no intervention (100/172, 58.1%), received attention control (eg, basic version of an app; 45/172, 26.2%), or were waitlisted (27/162, 15.7%). The majority had follow-up periods of 6 months or less (143/172, 83.1%), and only 2.3% (4/172) of studies had follow-up periods longer than 12 months. Over half reported a sample size calculation or power analysis (103/172, 59.9%) or an intention-to-treat analysis (96/172, 55.8%).

### Interventions

Over half of the interventions were delivered by a stand-alone app (106/172, 61.6%), and 38.4% (66/172) used an app as a component of a larger intervention. For example, 22 evaluated an app and in-person set-up (22/172, 12.8%),19 apps were paired with a wearable (19/172, 11.0%),15 evaluated an app with 2 other interventions (15/172, 8.7%), and 5.8% (10/172) apps had one other component that was neither in-person nor wearable (eg, text messages, mHealth support specialist). In total, 67 of the studies (67/172, 39.0%) reported that their intervention was based on a behavior change theory (eg, Social Cognitive Theory). One-size-fits-all (74/172, 43.0%) and dynamic (75/172, 43.6%) were the most common levels of personalization. Intervention frequency was mostly daily or multiple times per day (123/172, 71.5%). The studies assessed interventions for a range of behavior change outcomes. The top 3 were nutrition, physical activity, or both (60/172, 34.9%), mental health disorder management (34/172, 19.8%), and diabetes management (21/172, 12.2%).

### App Features

**Figure 3:**
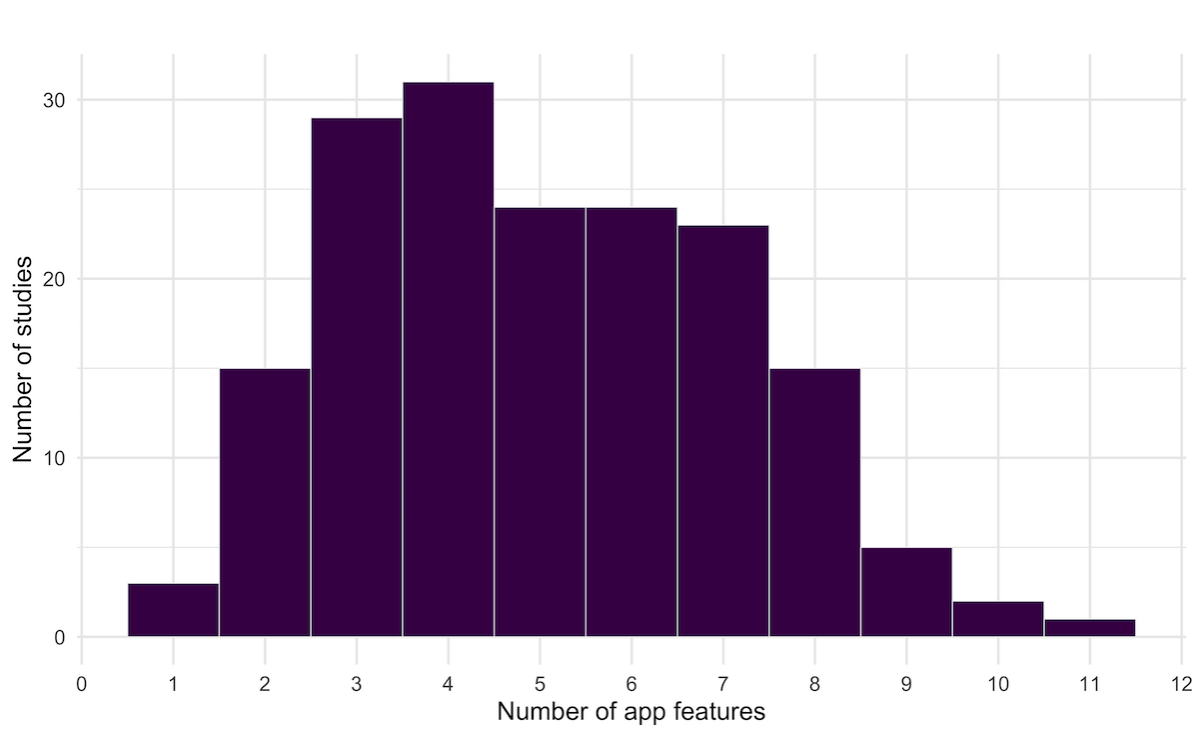
Number of studies with app features and frequency of app features.

**Figure 4:**
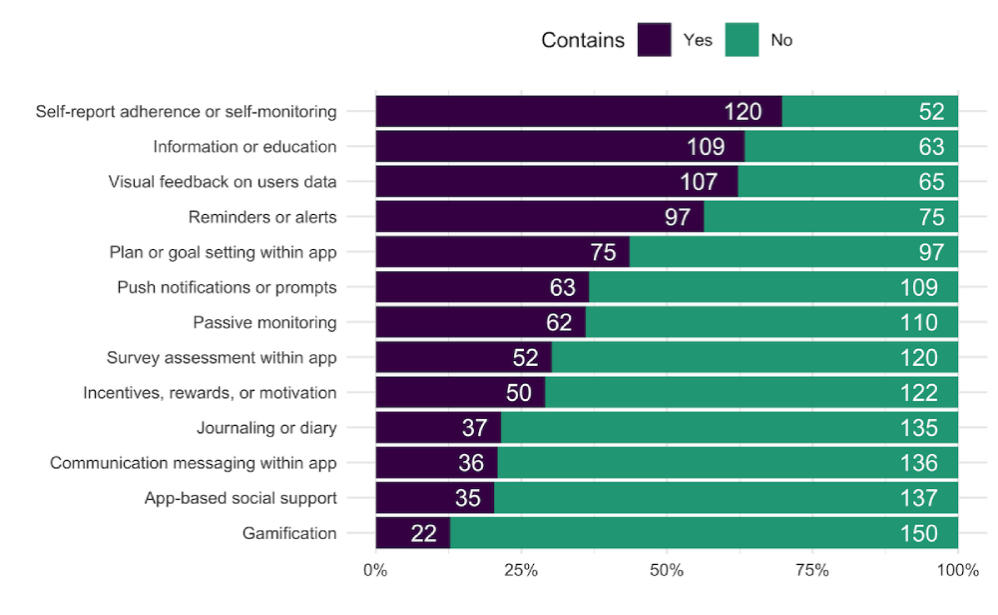
Frequency of app features.

The intervention apps included a mean of 5 features (SD 2), ranging from 1 to 11 (Figure 3). The most common features included self-report adherence or self-monitoring (120/172, 69.8%), visual feedback on user data (109/172, 63.4%), and information/education (107/172, 62.2%; Figure 4). The least common features were communication messaging within app (36/172, 20.9%), app-based social support (35/172, 20.3%), and gamification (22/172, 12.8%). There were no features common to all intervention apps. The features corresponded to one or more behavior change techniques (Multimedia Appendix 2). The most common behavior change mechanisms of the app features were feedback and monitoring (corresponding to 5 out of 13 features) and shaping knowledge (corresponding to 2 out of the 13 features).

### Risk of Bias

**Figure 5:**
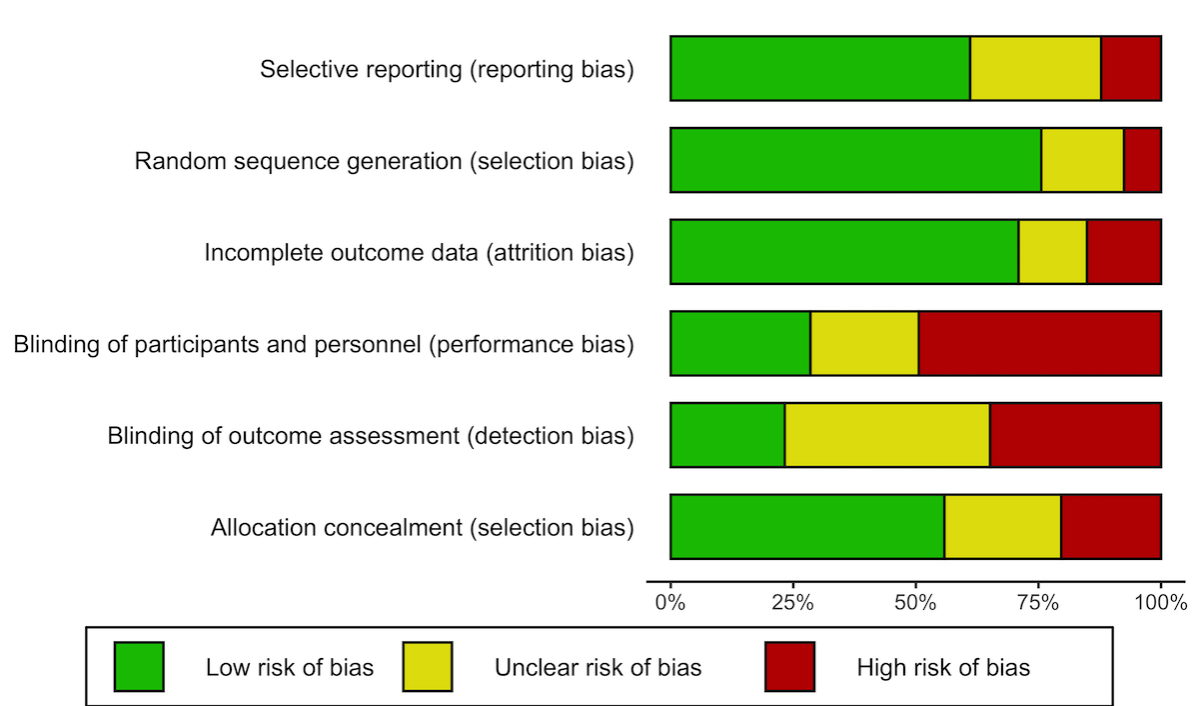
Risk of Bias.

All studies were screened for risk of bias using the Cochrane Risk of Bias tool for RCTs (Figure 5). The randomization procedure (random sequence generation) for most of the studies was considered adequately described (130/172, 75.6% low risk for bias). Just over half of studies reported details of allocation concealment (96/172, 55.8% had low risk of bias), whereas blinding of participants/personnel was less well described (49/172, 28.5% had low risk of bias). Few studies reported blinding of outcomes (40/172, 23.3%). Most studies had low risk of incomplete outcome data (122/172, 70.9%) and low risk of reporting bias (105/172, 61.0%).

### Meta-analysis

#### Overall Effect of Studies With Continuous Outcomes

**Figure 6:**
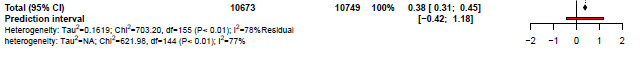
Effect of studies with continuous outcomes.

A total of 156 studies involving 21,422 participants reported continuous health outcome data. The use of a behavior change app (either as a stand-alone or as part of a larger intervention) confers a slight or weak advantage over standard care in health interventions (SMD=0.38 [95% CI 0.31-0.45]; I^2^=80%; Figure 6; Multimedia Appendices 3 and 4). Excluding pilot studies, there were a total of 105 studies involving 18,514 participants. The overall results were similar (SMD=0.35 [95% CI 0.27-0.43]; I^2^=82%).

#### Subanalyses of Studies With Continuous Outcomes

We analyzed the impact of the app level of personalization as stand-alone or as part of larger intervention and length of follow-up time. No clear pattern of relationship emerged in addition to the positive but weak advantage over standard care in health interventions for all groupings (Multimedia Appendix 3). Two groupings (>6 months, one-size-fits-all/static, part of larger intervention; <3 months/dynamic/stand-alone) had larger effect sizes, with SMD of 0.50 and 0.59, respectively. Excluding pilot studies in the analysis did not change the results.

### Publication Bias

**Figure 7:**
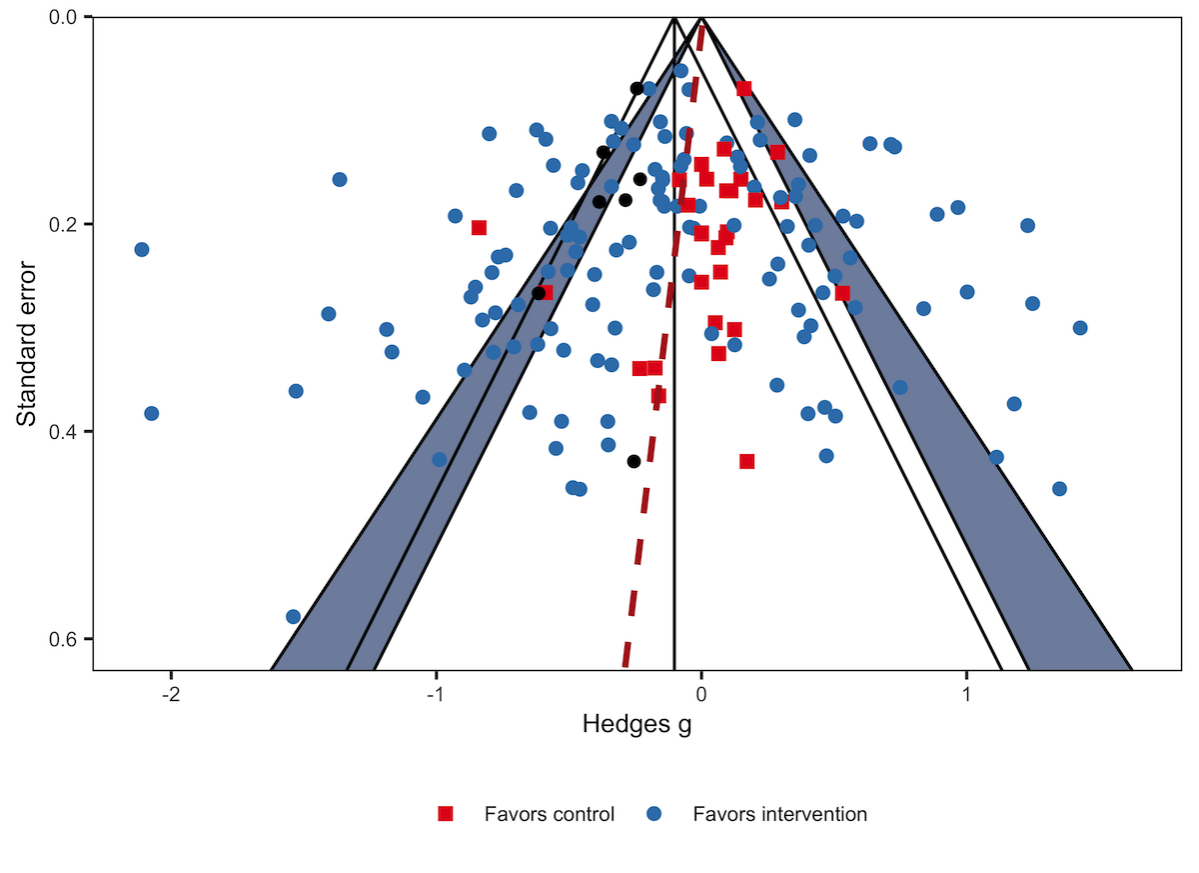
Funnel plot.

Figure 7 presents a funnel plot assessing publication bias for continuous outcomes. The trim-and-fill process was used to adjust for funnel plot asymmetry and estimate where studies might be without publication bias (black dots in Figure 7).

### Overall Effect of Studies With Binary Results

**Figure 8:**
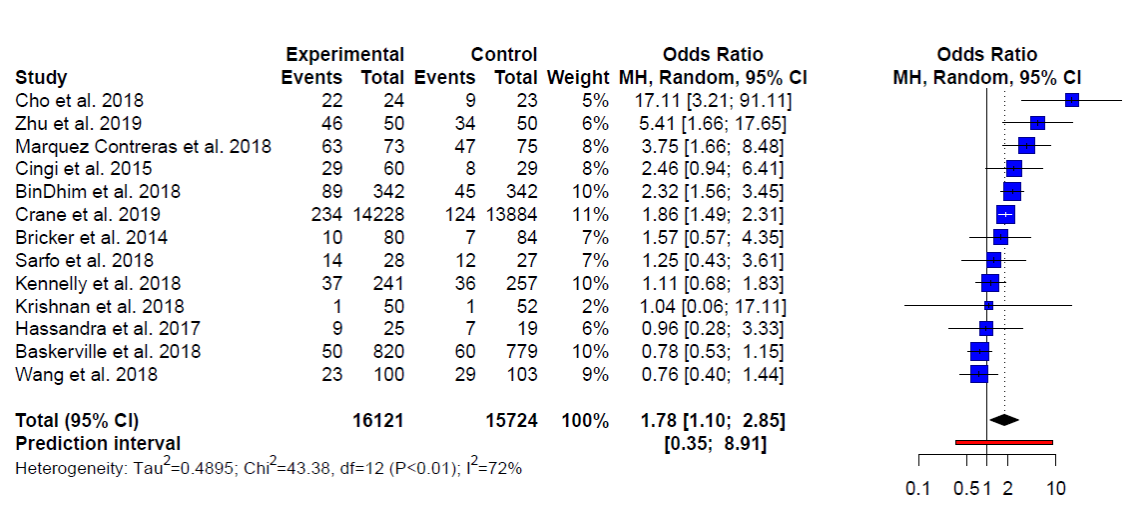
Effect of studies with binary outcomes.

A total of 13 studies involving 31,845 participants reported binary health outcome data. The use of a behavior change app (either as a stand-alone or as part of a larger intervention) confers a small advantage over standard care in health interventions with an odds ratio of 1.78 (95% CI 1.10-2.85; Figure 8).

Due to the small number of this group of studies, no subanalysis was conducted. We also did not assess publication bias in this small group of studies.

### Analysis of App Features of Successful Apps

**Figure 9:**
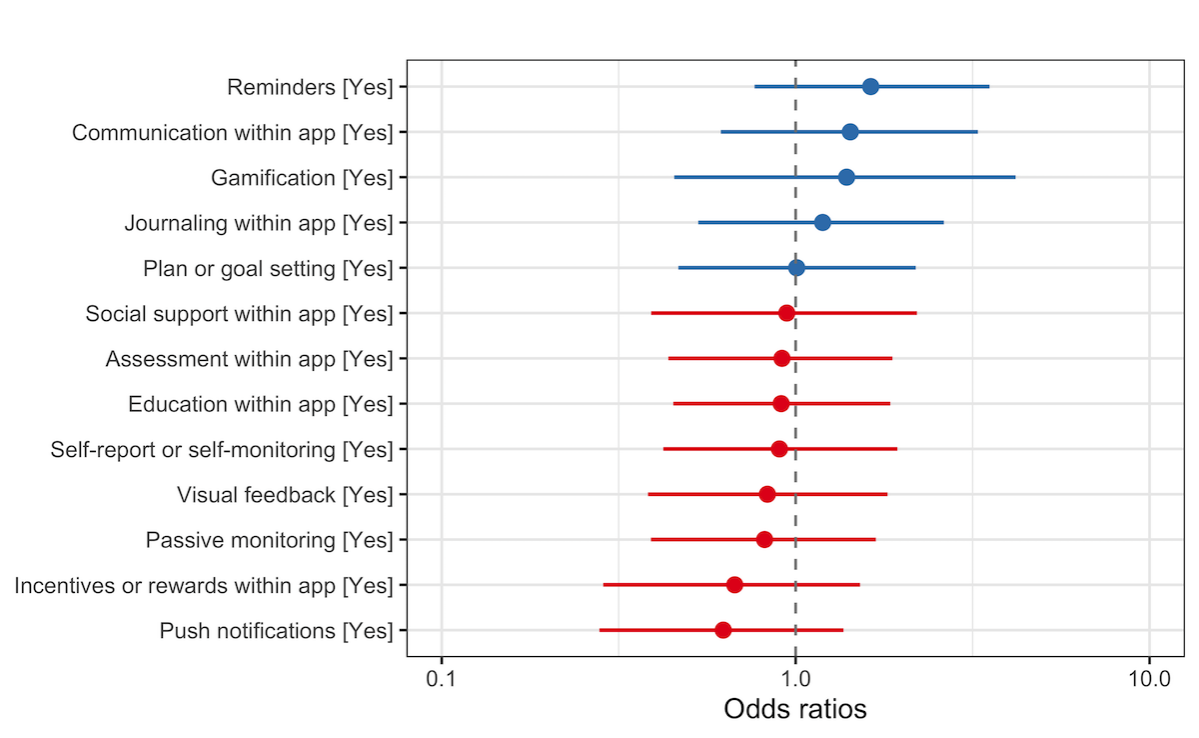
Effect of app features on outcome.

Results of logistic regression showed a slight positive effect on health outcomes for the features of interactive communication, reminders, gamification, and journaling, although no feature had significant effects on health outcomes (Figure 9).

## Discussion

### Principal Findings

In this comprehensive systematic review and meta-analysis, we synthesized the evidence from 172 studies, which included 53,331 participants, to describe the overall effect of mHealth apps used in interventions to change behavior to promote health and manage disease and also explored the impact of their features. To the best of our knowledge, this is the first meta-analysis to assess the effectiveness of health apps across a variety of diseases and health outcomes. Other related systematic reviews focused on methodologic reporting of any mHealth-based intervention (not app specific) [7], reported on specific diseases or health outcomes [12,15,18-21], explored features (not a systematic review) [34], or did not extract data for a meta-analysis [35,36].

Our results highlight a substantial growth in the number of health apps containing behavior change techniques being rigorously evaluated. In addition to the studies presented, 464 published protocols likely to meet the inclusion criteria were identified in the search, suggesting that the number of app-related RCT publications will likely continue to rise. The results from this meta-analysis suggest that app-based behavior change interventions have a positive, although weak, effect on health outcomes.

It is thought that mobile tools, such as apps, can significantly improve the provision of health care services and communication, particularly in low- and middle-income countries (LMICs) with a shortage of health care workers but where access to phones and cellular services is high even among vulnerable populations [37]. However, because the vast majority of studies included in this systematic review were conducted in high-income countries, the findings cannot be generalized to LMICs. Similarly, other systematic reviews of mHealth literature found disproportionate testing of mHealth tools, including apps, in high-income countries compared with LMICs [12,37-39].

### Quality of Selected Studies

We found the current evidence to be largely composed of studies reporting rigorous methodologies that included power calculations, intention-to-treat analysis, and a low risk of bias for randomization and randomization concealment. Most of the studies were not classified as pilot RCTs, but even with RCT pilot studies removed, results were largely unchanged. Randomized trials, if sufficiently large, provide the most convincing evidence about the effects of interventions because randomization should result in both observed and unobserved baseline characteristics being similar across the compared groups [27]. The high risk of bias for intervention blinding and blinding of outcomes arises from the near impossibility for patients and health care professionals to be unaware of the use of apps and smartphones in the care process. About 40% of the studies chose to use attention control or waitlist as comparators. Having an active comparator (eg, attention control) could ensure that all participants in a trial will not be knowingly disadvantaged and may reduce bias attributed to inadequate blinding [35].

### Overall Effect

Our finding of an overall positive but weak effect appears to be consistent with the few systematic reviews of app-based interventions that included meta-analyses. Systematic reviews and meta-analyses of apps targeting diabetes [12,21,40] and asthma control [18] found effects favoring app-based treatment. The weak effect could be due, in part, to high heterogeneity among the health outcomes. However, even the targeted reviews assessing the efficacy of apps for lifestyle modification in diabetes reported high heterogeneity of up to 86% in subcategory analysis [21,40], whereas the meta-analysis of apps to support asthma management included only 3 studies [18]. Behavior change interventions are inherently complex, and it is uncommon for any 2 interventions to evaluate exactly the same intervention [21]. Moreover, outcomes may be measured in different ways and at different time points across studies, which could further blur any differences in outcomes. We expected heterogeneity given the nature of behavior change interventions and the diversity of the disease outcomes studied. Nonetheless, the overall meta-analysis results in this study should be interpreted with caution because of the high heterogeneity found. Another contributing factor to the weak positive effect could be that a few studies reported the use of a behavior change theory to guide app development, which is recommended to improve the impact of an intervention [41]. Similarly, other researchers who conducted a systematic review of health apps noted a lack of mention by study authors of the use of a behavioral model or theory for the development of apps [42,43]. Along these same lines, we did not assess the contributions of patients and health care professionals in the development of app-based interventions, even though this factor is recognized as important [44]. It is well established that usability considerations along with the use of sociotechnical design principles and a holistic approach to behavior change can impact efficacy [42]. The involvement of patients and health care professionals in app development was not consistently mentioned by the authors of the studies included in this review. Finally, publication bias could undermine even the weak effect found. Negative or null findings are less often published or reported [45], and in this review, there appears to be publication bias toward studies that showed an intervention effect. However, funnel plots are not necessarily trustworthy under high heterogeneity. Nevertheless, as health behavior change interventions are resource intensive and often difficult to scale, having an equivalent or comparable outcome may still provide wider reach, cost savings, and added convenience to the users [2].

### Follow-Up Duration, Intervention Type, and Level of Personalization

We conducted sensitivity analysis on the length of follow-up time, the intervention as a stand-alone app or as part of a larger intervention, and the level of personalization. Sensitivity analyses suggested that the effects did not differ consistently based on these 3 attributes. We were concerned that the impact on effect could be skewed because the majority of the studies had follow-up times of less than 6 months. Although there was a slight trend for higher effects at lower follow-up times (less than 3 months), this was not consistent across all subanalyses groupings. Researchers have questioned the ability of app-based interventions to sustain beneficial health effects over time [40]. In particular, decline in app usage over time has been reported and could impact long-term effects [31]. In a systematic review of technology-driven behavior change techniques, researchers found that 63% were effective in the short term (<3 months), whereas only 33% were effective for long term (≥12 months) [46]. As most of the included studies targeted chronic conditions, more time may be needed to achieve a sustained behavior change that will lead to lasting changes in health outcomes. Our results are consistent with other research findings that short duration of follow-up was a limitation of many trials [7,11,13]. Another noted limitation of mHealth behavior change interventions is the lack of information on the impact of long-term intervention on patient-important primary outcomes [7]. Therefore, future intervention studies incorporating behavior change apps should increase follow-up times to enable assessment of primary outcomes as well as better describe app engagement overtime.

Regarding intervention type, it appeared that there was little impact on the overall effect if the intervention was provided as a stand-alone app or if the app was part of a larger intervention. This is unsurprising as behavior change interventions are typically complex, include multiple components, and may impact individuals in a variety of ways [7]. In the studies in which the app formed part of a larger intervention, other components included, for example, occasional in-person meetings or phone calls, a wearable device, or a combination. As the stand-alone apps had comparable results with interventions that included other components, this may suggest that apps can support a comprehensive intervention.

The level of personalization, either one-size-fits-all or static or dynamic, did not appear to alter the overall effect of outcomes. This runs counter to recommendations to offer levels of personalization rather than a one-size-fits-all functionality to improve outcome [47]. For instance, a systematic review of app features for diabetic support concluded that personalized and tailored empowerment features should be included in commercial apps for large-scale assessment of the self-management of the disease [48].

### Contribution of Features

In this study, the number of features present in the app did not appear to confer an advantage. This contrasts with other researchers who reported increased efficacy with more features [12,46]. The impact of the features on health-related outcomes was inconsistent [18]. It has been proposed that an increase in the number of features could decrease the usability of an app [44]. Usability is considered critical for engagement and adherence to app interventions over time [49]. Further exploration of the complex interaction between the number of features and usability may be important for determining future app efficacy. In addition, future research could focus on better understanding of mechanisms of action of behavior change techniques within health app–based interventions to assess which combinations lead to improved health outcomes. Our aim was not to specifically detect which behavior change techniques could be the mechanism of action for the potential changes in health outcomes. The authors of app-based studies should clearly describe the behavior change techniques used in their app components and the guiding theories. Applying taxonomy of behavior change techniques by Michie et al [24] could be used to help compare and contrast findings across studies. However, these taxonomies were not developed for coding app features; therefore, some concepts may not be fully transferable [5]. Features represent a high level of abstraction of requirements and are useful to describe the functionality of a new system without the need to drill down into too much detail or are later specific enough for implementation [50]. Research is needed to better tailor and align behavior change taxonomies for mobile app feature apps.

When individual features were assessed by health outcome effectiveness, there appeared to be a slight advantage among those apps using interactive communication, reminders, gamification, and journaling. Interactive communication has been found to be important in a number of studies. Among individuals with diabetes, including interactive communication in apps and having remote access to health professionals were associated with greater effectiveness in reducing hemoglobin A_1C_ levels [12]. Similarly, two-way interactivity has been shown to improve adherence [51]. Furthermore, smartphone-based interventions that did not include interactive support saw a decrease in app use/engagement among those with chronic respiratory diseases, diabetes, and hypertension [34]. Reminders have also been identified as a core component of mobile interventions, most notably through the use of text messaging interventions [52].

Gamification conferred an advantage even though it was the least frequent feature type. Identifying few apps that employed gamification is consistent with findings from other studies, in which authors reported that this lack of use may limit the potential to improve health outcomes [53,54]. Journaling as a feature was not mentioned in other systematic reviews of features; thus, it may be a new finding or may have been labeled differently. In any type of technology-driven intervention for diabetes management, those containing digital features that facilitated health and lifestyle education, behavior or outcome tracking, and/or web-based health coaching were most effective [46]. In this review, we identified many studies that included education, self-tracking, coaching, and goal setting; however, they did not show a clear effect on health outcomes.

### Strengths and Limitations

A strength of this study is that it is a comprehensive review of rigorously tested app-delivered behavior change interventions for a variety of diseases and health outcomes. Understanding the impact of app-delivered interventions and features is important given their rapid growth in the health care market. There are also limitations to consider. First, although we attempted to focus on stand-alone app interventions, some of the studies included other elements with the apps used as one component of a larger intervention; thus, it was difficult to isolate the effects of app-based interventions. We attempted to mitigate this limitation by conducting subanalyses with stand-alone apps. Second, we did not extract data on app usage. Usage data could elucidate whether the intervention was less effective because of lack of use or exposure to the intervention. However, these data were not consistently reported in the studies. Finally, there was often a lack of clear reporting of app features, which made it challenging to extract data. If the CONSORT-EHEALTH (Consolidated Standards of Reporting Trials of Electronic and Mobile HEalth Applications and onLine TeleHealth) guideline continues to be adopted for mHealth research, reporting quality should begin to improve [55,56].

### Future Recommendations

Future mHealth studies should use standardized reporting guidelines and describe their intervention and app features with adequate detail so that results are reproducible [56]. Future research is needed to develop a taxonomy of behavior change techniques that aligns with all potential app features. These efforts have begun with focus on specific app types, such as measuring physical activity [57,58] and reducing alcohol consumption [59]. Follow-up times should be appropriate for disease and condition and likely should be longer than what we found in this study, where the majority of papers reported follow-up durations of less than 6 months. Updates to this review can be conducted to assess progress as the published protocols are completed and reported. In addition to understanding clinical effectiveness and cost-effectiveness, future work will also need to include the potential payer’s perspective to lessen administrative burden, improve workflow, enhance patient and provider engagement, and improve quality of care while lowering costs [16]. Adoption or adherence to the technology should also be evaluated.

### Conclusions

Rigorous studies to examine the effectiveness of behavior change app interventions on health outcomes are increasing. The results of our meta-analysis suggest that apps have a positive but weak effect on health outcomes and may be a useful adjunct in behavior change health interventions. There was insufficient evidence to make recommendations on the essential number of base features to include in apps. There is a clear need for rigorous testing of behavior change apps in LMICs where there may be added challenges of lack of human resources and access to health care services. Future research should clearly report app features, evaluate long-term effectiveness to modify health outcomes, and consider attention control comparators. In addition, negative or null findings need to be reported. Although not explored in this review, including analyses of level of app engagement is needed to better determine the actual effect of apps on outcomes and to explore the specific features that promote patient engagement with the app and adherence to the intervention.

## Appendix

Multimedia Appendix 1 MEDLINE (Ovid) Search Strategy.

Multimedia Appendix 2 App features and definitions.

Multimedia Appendix 3 Effects of studies with continuous outcomes by level of personalization, intervention type, and length of outcome follow-up.

Multimedia Appendix 4 List of references cited in Multimedia Appendix 3.

## Abbreviations

LMIC: low- and middle-income country
mHealth: mobile health
PRISMA: Preferred Reporting Items for Systematic Reviews and Meta-Analysis
RCT: randomized controlled trial
SMD: standardized mean difference
