# Supplementary material for: Effectiveness of Mobile Apps to Promote Health and Manage Disease: Systematic Review and Meta-analysis of Randomized Controlled Trials
Source: JMIR Mhealth Uhealth. 2021 Jan 11;9(1):e21563. doi: 10.2196/21563 (PMC7834932; doi:10.2196/21563)
Supplement: Multimedia Appendix 1 [file mhealth_v9i1e21563_app1.docx]

Appendix 1. MEDLINE (Ovid) Search Strategy

| 1 | ((mobile or portable electronic or portable software or smartphone* or smart-phone* or cellphone* or "cell phone" or "cellular phone" or tablet* or ipad* or ipod* or android or nexus or iOS or handheld* or hand-held* or notebook* or "note book*") adj3 (apps or app or application or applications)).mp. [mp=title, abstract, original title, name of substance word, subject heading word, keyword heading word, protocol supplementary concept word, rare disease supplementary concept word, unique identifier] |
| --- | --- |
| 2 | (Telemedicine/ or (telehealth or tele-health or telemedicine or tele-medicine or mhealth or m-health or ehealth or e-health or "mobile application" or "mobile applications" or "mobile apps" or "mobile app" or application or applications or app or apps).mp.) and (exp Cell phones/ or ((mobile or cell or cellular or smart) adj3 (phones or phone)).mp. or smartphone*.mp. or tablet.mp. or tablets.mp. or iPad*.mp. or iPod*.mp. or nexus.mp. or android.mp. or ios.mp.) [mp=title, abstract, original title, name of substance word, subject heading word, keyword heading word, protocol supplementary concept word, rare disease supplementary concept word, unique identifier] |
| 3 | exp Mobile Applications/ |
| 4 | 1 or 2 or 3 |
| 5 | (((((chronic or infectious or communicable or "long term" or longterm) adj3 (illness* or disease* or condition*)) or "chronically ill").mp. or exp Chronic Disease/ or exp Disease Management/ or disease.mp.) adj3 management.mp.) or exp Communicable Diseases/ [mp=title, abstract, original title, name of substance word, subject heading word, keyword heading word, protocol supplementary concept word, rare disease supplementary concept word, unique identifier] |
| 6 | exp Arthritis/ or (arthritis or Arthritides or Polyarthritis or Polyarthritides).mp. or exp Obesity/ or obesity.mp. or obese.mp. or exp Heart Diseases/ or "heart disease*".mp. or "cardiac disease*".mp. or exp Neoplasms/ or (cancer or neoplasm* or tumor* or neoplasia or malignan*).mp. or exp epilepsy/ or (epileps* or seizure* or epileptic).mp. or exp "Tobacco Use"/ or (smoking or tobacco).mp. |
| 7 | exp Diabetes Mellitus/ or (diabetes or diabetic or "glucose intolerance").mp. or exp hypertension/ or hypertensi*.mp. or ((high or elevated or increased) adj3 "blood pressure*").mp. or exp asthma/ or asthma.mp. or exp pulmonary disease chronic obstructive/ or (chronic adj2 obstructive adj2 pulmonary).mp. or COPD.mp. or exp thyroid diseases/ or (thyroid adj3 disease*).mp. |
| 8 | exp Hyperlipidemias/ or (Hyperlipemia* or Hyperlipidemia* or lipemia* or lipiemia*).mp. or exp mental disorders/ or ((mental or behavior or psychiatric) adj3 disorder*).mp. |
| 9 | (anxiety or bipolar or Schizophrenia or depression).mp. or exp Depression/ or exp Substance-Related Disorders/ or "drug dependence".mp. or (substance adj3 (disorder* or abuse or dependence or addiction)).mp. or (drug adj3 (disorder* or abuse or dependence addiction)).mp. or exp HIV infections/ or (HIV or AIDS).mp. or ("cystic fibrosis" or osteoporosis or (crohn* adj3 disease) or lupus or hepatitis or endometriosis or "sickle cell" or fibromyalgia or "multiple sclerosis").mp. |
| 10 | 5 or 6 or 7 or 8 or 9 |
| 11 | (outcome* or readmission* or adhere* or complian* or cooperation or noncomplian* or effective* or improve* or intervention* or assess* or prognosis or (health adj3 behavior*)).mp. or exp Health Behavior/ |
| 12 | 4 and 10 and 13 |
| 13 | (eng or spa).lg. |
| 14 | 14 and 15 |
| 15 | limit 14 to yr="2006 -Current" |
