# Supplementary material for: Effectiveness of Mobile Apps to Promote Health and Manage Disease: Systematic Review and Meta-analysis of Randomized Controlled Trials
Source: JMIR Mhealth Uhealth. 2021 Jan 11;9(1):e21563. doi: 10.2196/21563 (PMC7834932; doi:10.2196/21563)
Supplement: Multimedia Appendix 2 [file mhealth_v9i1e21563_app2.docx]

**Appendix 2. App feature, corresponding behavior change technique, definition, and examples**

| **Feature** | **Behavior change technique[1]** | **Definition** | **Examples** |
| --- | --- | --- | --- |
| Tracking / self-monitoring | Feedback and Monitoring (2.2) | Active self-monitoring or reporting by recording information in order to modify personal attitudes or behaviors | track symptoms, adherence |
| Reminders/alert | Feedback and Monitoring (2.2) | Prompts the user to partake in a specific behavior using a predetermined alert | Medication reminder |
| Push notification or prompt | Associations – prompts/cues (7.1) | pops up messages on mobile device; users don't have to be in the app or using their devices to receive them | message, pop-up, can be a reminder, motivation |
| Gamification | Reward and Threat – incentive (10.1) | Offers points, badges, or movement through levels as a health objective is achieved or as more engaged | Points, scores |
| Survey/ assessment | Feedback and monitoring – self-monitoring of behavior (2.3) | Within app survey, screening, or questionnaire | respond to questions or survey of behavior |
| Communication / messaging | Shaping knowledge, social support (4.3, 3.1) | Bi-directional communication within app with person/health provider | Coaching or chat bot |
| Information/ Education | Shaping knowledge (4.3) | Basic education material about a disease/condition including causes, treatment or management | Frequently asked questions and answers |
| Social support | Social support (3.2) | Access to disease specific discussion boards, connects users to app communities where consumers and family/caregivers can communicate via posts and private messages, or external social support | Intervention specific Facebook page |
| Passive Monitoring | Feedback and Monitoring (2.2) | Passive tracking of personal data | steps, BG, BP, weigh |
| Goal planning / Tailored recommendation | Goals and planning (1.1, 1.3) | Plan or goal setting or tailored recommendations within app for behavior change | personalized quit smoking plan, weight loss plan |
| Incentive/ Reward | Reward and threat (10.1. 10.3) | Incentive, rewards, motivation/ motivational messages |  |
| Visual Feedback | Feedback and monitoring (2.6) | Provides feedback or progress information based on inputted data | adherence record, graph of steps over time |
| Journaling/diary | Self-belief (15.3), Goals and planning (1.1, 1.3) | Prompts users to record notes and experiences or responses to intervention | pain management efforts |

1. Michie S, W.C., Johnston M, et al., *Behaviour change techniques: the development and evaluation of a taxonomic method for reporting and describing behaviour change interventions (a suite of five studies involving consensus methods, randomised controlled trials and analysis of qualitative data)*. Health Technology Assessment. Vol. 19.99. 2015, Southampton (UK) NIHR Journals Library.
