# Supplementary figures and images for: Effectiveness of Mobile Apps to Promote Health and Manage Disease: Systematic Review and Meta-analysis of Randomized Controlled Trials
Source: JMIR Mhealth Uhealth. 2021 Jan 11;9(1):e21563. doi: 10.2196/21563 (PMC7834932; doi:10.2196/21563)

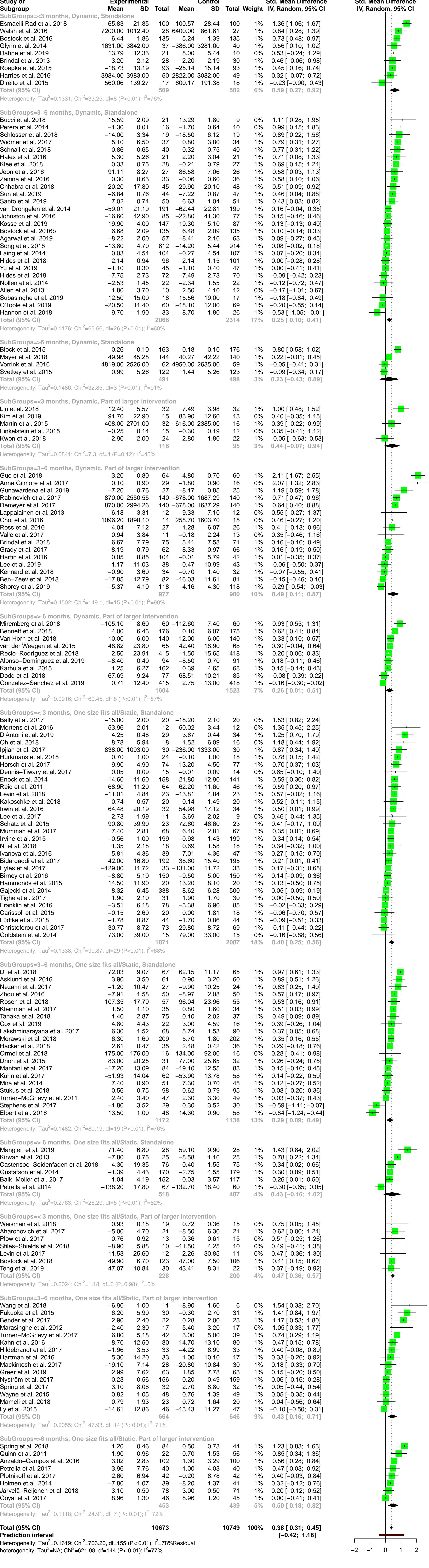

Supplement: Multimedia Appendix 3 [file mhealth_v9i1e21563_app3.pdf]
