# Supplementary material for: Effectiveness of Mobile Apps to Promote Health and Manage Disease: Systematic Review and Meta-analysis of Randomized Controlled Trials
Source: JMIR Mhealth Uhealth. 2021 Jan 11;9(1):e21563. doi: 10.2196/21563 (PMC7834932; doi:10.2196/21563)
Supplement: Multimedia Appendix 4 [file mhealth_v9i1e21563_app4.docx]

## **Multimedia Appendix 4: List of references cited in Multimedia Appendix 3.**

| Authors | Year | Title |
| --- | --- | --- |
| Agarwal, P.; Mukerji, G.; Desveaux, L.; Ivers, N. M.; Bhattacharyya, O.; Hensel, J. M.; Shaw, J.; Bouck, Z.; Jamieson, T.; Onabajo, N.; Cooper, M.; Marani, H.; Jeffs, L.; Bhatia, R. S. | 2019 | Mobile App for Improved Self-Management of Type 2 Diabetes: Multicenter Pragmatic Randomized Controlled Trial |
| Aharonovich, E.; Stohl, M.; Cannizzaro, D.; Hasin, D. | 2017 | HealthCall delivered via smartphone to reduce co-occurring drug and alcohol use in HIV-infected adults: A randomized pilot trial |
| Allen, Jerilyn K.; Stephens, Janna; Dennison Himmelfarb, Cheryl R.; Stewart, Kerry J.; Hauck, Sara | 2013 | Randomized controlled pilot study testing use of smartphone technology for obesity treatment |
| Alonso-Domínguez, R.; Garci­a-Ortiz, L.; Patino-Alonso, M. C.; SÃ¡nchez-Aguadero, N.; GÃ³mez-Marcos, M. A.; Recio-RodrÃ­guez, J. I. | 2019 | Effectiveness of a multifactorial intervention in increasing adherence to the mediterranean diet among patients with diabetes mellitus type 2: A controlled and randomized study (EMID study) |
| Anne Gilmore, L.; Klempel, M. C.; Martin, C. K.; Myers, C. A.; Burton, J. H.; Sutton, E. F.; Redman, L. M. | 2017 | Personalized Mobile Health Intervention for Health and Weight Loss in Postpartum Women Receiving Women, Infants, and Children Benefit: A Randomized Controlled Pilot Study |
| Anzaldo-Campos, Maria Cecilia; Contreras, Sonia; Vargas-Ojeda, Adriana; Menchaca-Diaz, Rufino; Fortmann, Addie; Philis-Tsimikas, Athena | 2016 | Dulce Wireless Tijuana: A Randomized Control Trial Evaluating the Impact of Project Dulce and Short-Term Mobile Technology on Glycemic Control in a Family Medicine Clinic in Northern Mexico |
| Asklund, I; Nystrom, E; Sjostrom, M; Umefjord, G; Stenlund, H; Samuelsson, E | 2016 | Mobile app for treatment of stress urinary incontinence: a randomized controlled trial |
| Balk-Moller, Nina Charlotte; Poulsen, Sanne Kellebjerg; Larsen, Thomas Meinert | 2017 | Effect of a Nine-Month Web- and App-Based Workplace Intervention to Promote Healthy Lifestyle and Weight Loss for Employees in the Social Welfare and Health Care Sector: A Randomized Controlled Trial |
| Bally, L; Dehais, J; Nakas, Ct; Anthimopoulos, M; Laimer, M; Rhyner, D; Rosenberg, G; Zueger, T; Diem, P; Mougiakakou, S; Stettler, C | 2017 | Carbohydrate estimation supported by the GoCARB system in individuals with type 1 diabetes: a randomized prospective pilot study |
| Baskerville, N. B.; Struik, L. L.; Guindon, G. E.; Norman, C. D.; Whittaker, R.; Burns, C.; Hammond, D.; Dash, D.; Brown, K. S. | 2018 | Effect of a Mobile Phone Intervention on Quitting Smoking in a Young Adult Population of Smokers: Randomized Controlled Trial |
| Ben-Zeev, D.; Brian, R. M.; Jonathan, G.; Razzano, L.; Pashka, N.; Carpenter-Song, E.; Drake, R. E.; Scherer, E. A. | 2018 | Mobile health (mHealth) versus clinic-based group intervention for people with serious mental illness: A randomized controlled trial |
| Bender, M. S.; Cooper, B. A.; Park, L. G.; Padash, S.; Arai, S. | 2017 | A Feasible and Efficacious Mobile-Phone Based Lifestyle Intervention for Filipino Americans with Type 2 Diabetes: Randomized Controlled Trial |
| Bennett, Gary G.; Steinberg, Dori; Askew, Sandy; Levine, Erica; Foley, Perry; Batch, Bryan C.; Svetkey, Laura P.; Bosworth, Hayden B.; Puleo, Elaine M.; Brewer, Ashley; DeVries, Abigail; Miranda, Heather | 2018 | Effectiveness of an app and provider counseling for obesity treatment in primary care |
| Bidargaddi, Niranjan; Musiat, Peter; Winsall, Megan; Vogl, Gillian; Blake, Victoria; Quinn, Stephen; Orlowski, Simone; Antezana, Gaston; Schrader, Geoffrey | 2017 | Efficacy of a Web-Based Guided Recommendation Service for a Curated List of Readily Available Mental Health and Well-Being Mobile Apps for Young People: Randomized Controlled Trial |
| BinDhim, N. F.; McGeechan, K.; Trevena, L. | 2018 | Smartphone Smoking Cessation Application (SSC App) trial: a multicountry double-blind automated randomised controlled trial of a smoking cessation decision-aid 'app' |
| Birney, Amelia J.; Gunn, Rebecca; Russell, Jeremy K.; Ary, Dennis V. | 2016 | MoodHacker Mobile Web App With Email for Adults to Self-Manage Mild-to-Moderate Depression: Randomized Controlled Trial |
| Block, Gladys; Azar, Kristen Mj; Romanelli, Robert J.; Block, Torin J.; Hopkins, Donald; Carpenter, Heather A.; Dolginsky, Marina S.; Hudes, Mark L.; Palaniappan, Latha P.; Block, Clifford H. | 2015 | Diabetes Prevention and Weight Loss with a Fully Automated Behavioral Intervention by Email, Web, and Mobile Phone: A Randomized Controlled Trial Among Persons with Prediabetes |
| Bostock, S; Luik, Ai; Espie, Ca | 2016 | Sleep and Productivity Benefits of Digital Cognitive Behavioral Therapy for Insomnia: a Randomized Controlled Trial Conducted in the Workplace Environment |
| Bostock, S.; Crosswell, A. D.; Prather, A. A.; Steptoe, A. | 2018 | Mindfulness On-The-Go: Effects of a Mindfulness Meditation App on Work Stress and Well-Being |
| Bostock, Sophie PhD; Luik, Annemarie I. PhD; Espie, Colin A. PhD | 2016 | sleep and Productivity Benefits of Digital Cognitive Behavioral Therapy for Insomnia: a Randomized Controlled Trial Conducted in the Workplace Environment |
| Bricker, Jonathan B.; Mull, Kristin E.; Kientz, Julie A.; Vilardaga, Roger; Mercer, Laina D.; Akioka, Katrina J.; Heffner, Jaimee L. | 2014 | Randomized, controlled pilot trial of a smartphone app for smoking cessation using acceptance and commitment therapy |
| Brindal, E.; Hendrie, G. A.; Freyne, J.; Noakes, M. | 2018 | Incorporating a static versus supportive mobile phone app into a partial meal replacement program with face to face support, a randomized controlled trial |
| Brindal, Emily; Hendrie, Gilly A.; Taylor, Pennie; Freyne, Jill; Noakes, Manny | 2016 | Cohort Analysis of a 24-Week Randomized Controlled Trial to Assess the Efficacy of a Novel, Partial Meal Replacement Program Targeting Weight Loss and Risk Factor Reduction in Overweight/Obese Adults |
| Brindal, Emily; Hendrie, Gilly; Freyne, Jill; Coombe, Mac; Berkovsky, Shlomo; Noakes, Manny | 2013 | Design and pilot results of a mobile phone weight-loss application for women starting a meal replacement programme |
| Bucci, S.; Barrowclough, C.; Ainsworth, J.; Machin, M.; Morris, R.; Berry, K.; Emsley, R.; Lewis, S.; Edge, D.; Buchan, I.; et al. | 2018 | Actissist: proof-of-Concept Trial of a Theory-Driven Digital Intervention for Psychosis |
| Carissoli, C; Villani, D; Riva, G | 2015 | Does a meditation protocol supported by a mobile application help people reduce stress? Suggestions from a controlled pragmatic trial |
| Castensoe-Seidenfaden, P.; Husted, G. R.; Jensen, A. K.; Hommel, E.; Olsen, B.; Pedersen-Bjergaard, U.; Kensing, F.; Teilmann, G. | 2018 | Testing a Smartphone App (Young with Diabetes) to Improve Self-Management of Diabetes Over 12 Months: Randomized Controlled Trial |
| Chhabra, H. S.; Sharma, S.; Verma, S. | 2018 | Smartphone app in self-management of chronic low back pain: a randomized controlled trial |
| Cho, S. W.; Wee, J. H.; Yoo, S.; Heo, E.; Ryu, B.; Kim, Y.; Lee, J. S.; Kim, J. W. | 2018 | Effect of Lifestyle Modification Using a Smartphone Application on Obesity With Obstructive Sleep Apnea: A Short-term, Randomized Controlled Study |
| Choi, JiWon; Lee, Ji hyeon; Vittinghoff, Eric; Fukuoka, Yoshimi | 2016 | mHealth physical activity intervention: A randomized pilot study in physically inactive pregnant women |
| Christoforou, M.; Saez Fonseca, J. A.; Tsakanikos, E. | 2017 | Two Novel Cognitive Behavioral Therapy-Based Mobile Apps for Agoraphobia: Randomized Controlled Trial |
| Cingi, Cemal; Yorgancioglu, Arzu; Cingi, Can Cemal; Oguzulgen, Kivilcim; Muluk, Nuray Bayar; Ulusoy, Seckin; Orhon, Nezih; Yumru, Cengiz; Gokdag, Dursun; Karakaya, Gul; Celebi, Saban; Cobanoglu, H. Bengu; Unlu, Halis; Aksoy, Mehmet Akif | 2015 | The "physician on call patient engagement trial" (POPET): measuring the impact of a mobile patient engagement application on health outcomes and quality of life in allergic rhinitis and asthma patients |
| Cox, C. E.; Hough, C. L.; Jones, D. M.; Ungar, A.; Reagan, W.; Key, M. D.; Gremore, T.; Olsen, M. K.; Sanders, L.; Greeson, J. M.; et al. | 2019 | Effects of mindfulness training programmes delivered by a self-directed mobile app and by telephone compared with an education programme for survivors of critical illness: a pilot randomised clinical trial |
| Crane, D.; Ubhi, H. K.; Brown, J.; West, R. | 2019 | Relative effectiveness of a full versus reduced version of the â€˜smoke freeâ€™ mobile application for smoking cessation: An exploratory randomised controlled trial [version 2; referees: 2 approved] |
| D'Antoni, D.; Auyeung, V.; Walton, H.; Fuller, G. W.; Grieve, A.; Weinman, J. | 2019 | The effect of evidence and theory-based health advice accompanying smartphone air quality alerts on adherence to preventative recommendations during poor air quality days: A randomised controlled trial |
| Dahne, J.; Collado, A.; Lejuez, C. W.; Risco, C. M.; Diaz, V. A.; Coles, L.; Kustanowitz, J.; Zvolensky, M. J.; Carpenter, M. J. | 2019 | Pilot randomized controlled trial of a Spanish-language Behavioral Activation mobile app (Aptivate!) for the treatment of depressive symptoms among united states Latinx adults with limited English proficiency |
| Demeyer, H.; Louvaris, Z.; Frei, A.; Rabinovich, R. A.; de Jong, C.; Gimeno-Santos, E.; Loeckx, M.; Buttery, S. C.; Rubio, N.; Van der Molen, T.; Hopkinson, N. S.; Vogiatzis, I.; Puhan, M. A.; Garcia-Aymerich, J.; Polkey, M. I.; Troosters, T.; Mr Papp, PROactive study group; the, PROactive consortium | 2017 | Physical activity is increased by a 12-week semiautomated telecoaching programme in patients with COPD: a multicentre randomised controlled trial |
| Dennis-Tiwary, Tracy A.; Denefrio, Samantha; Gelber, Shari | 2017 | Salutary effects of an attention bias modification mobile application on biobehavioral measures of stress and anxiety during pregnancy |
| Di, R.; Li, G. | 2018 | Use of a Smartphone Medical App Improves Complications and Quality of Life in Patients with Nasopharyngeal Carcinoma Who Underwent Radiotherapy and Chemotherapy |
| Direito, A; Jiang, Y; Whittaker, R; Maddison, R | 2015 | Apps for IMproving FITness and Increasing Physical Activity Among Young People: The AIMFIT Pragmatic Randomized Controlled Trial |
| Dodd, Jodie M.; Louise, Jennie; Cramp, Courtney; Grivell, Rosalie M.; Moran, Lisa J.; Deussen, Andrea R. | 2018 | Evaluation of a smartphone nutrition and physical activity application to provide lifestyle advice to pregnant women: The SNAPP randomised trial |
| Drion, Iefke; Pameijer, Loes R.; van Dijk, Peter R.; Groenier, Klaas H.; Kleefstra, Nanne; Bilo, Henk J. G. | 2015 | The Effects of a Mobile Phone Application on Quality of Life in Patients With Type 1 Diabetes Mellitus: A Randomized Controlled Trial |
| Elbert, Sp; Dijkstra, A; Oenema, A | 2016 | A Mobile Phone App Intervention Targeting Fruit and Vegetable Consumption: the Efficacy of Textual and Auditory Tailored Health Information Tested in a Randomized Controlled Trial |
| Enock, P. M.; Hofmann, S. G.; McNally, R. J. | 2014 | Attention bias modification training via smartphone to reduce social anxiety: A randomized, controlled multi-session experiment |
| Esmaeili Rad, M.; Ahmadi, F. | 2018 | A new method to measure and decrease the online social networking addiction |
| Eyles, Helen; McLean, Rebecca; Neal, Bruce; Jiang, Yannan; Doughty, Robert N.; McLean, Rachael; Ni Mhurchu, Cliona | 2017 | A salt-reduction smartphone app supports lower-salt food purchases for people with cardiovascular disease: Findings from the SaltSwitch randomised controlled trial |
| Finkelstein, J; Bedra, M; Li, X; Wood, J; Ouyang, P | 2015 | Mobile App to Reduce Inactivity in Sedentary Overweight Women |
| Franklin, Jc; Fox, Kr; Franklin, Cr; Kleiman, Em; Ribeiro, Jd; Jaroszewski, Ac; Hooley, Jm; Nock, Mk | 2016 | A brief mobile app reduces nonsuicidal and suicidal self-injury: Evidence from three randomized controlled trials |
| Fukuoka, Y.; Gay, C. L.; Joiner, K. L.; Vittinghoff, E. | 2015 | A Novel Diabetes Prevention Intervention Using a Mobile App |
| Gajecki, Mikael; Berman, Anne H.; Sinadinovic, Kristina; Rosendahl, Ingvar; Andersson, Claes | 2014 | Mobile phone brief intervention applications for risky alcohol use among university students: a randomized controlled study |
| Glynn, Liam G.; Hayes, Patrick S.; Casey, Monica; Glynn, Fergus; Alvarez-Iglesias, Alberto; Newell, John; Olaighin, Gearoid; Heaney, David; O'Donnell, Martin; Murphy, Andrew W. | 2014 | Effectiveness of a smartphone application to promote physical activity in primary care: the SMART MOVE randomised controlled trial |
| Goldstein, Carly M.; Gathright, Emily C.; Dolansky, Mary A.; Gunstad, John; Sterns, Anthony; Redle, Joseph D.; Josephson, Richard; Hughes, Joel W. | 2014 | Randomized controlled feasibility trial of two telemedicine medication reminder systems for older adults with heart failure |
| Gomez-Marcos, M. A.; Patino-Alonso, M. C.; Recio-Rodriguez, J. I.; Agudo-Conde, C.; Romaguera-Bosch, M.; Magdalena-Gonzalez, O.; Gomez-Arranz, A.; Mendizabal-Gallastegui, N.; Angel Fernandez-Diez, J.; Gomez-Sanchez, L.; Maderuelo-Fernandez, J. A.; Rodriguez-Sanchez, E.; Garcia-Ortiz, L.; on behalf the, Evident Investigators | 2018 | Short- and long-term effectiveness of a smartphone application for improving measures of adiposity: A randomised clinical trial - EVIDENT II study |
| Gonzalez-Sanchez, J.; Recio-Rodriguez, J. I.; Fernandez-delRio, A.; Sanchez-Perez, A.; Magdalena-Belio, J. F.; Gomez-Marcos, M. A.; Garcia-Ortiz, L.; On behalf the, Evident Investigators group | 2019 | Using a smartphone app in changing cardiovascular risk factors: A randomized controlled trial (EVIDENT II study) |
| Goyal, Shivani; Nunn, Caitlin A.; Rotondi, Michael; Couperthwaite, Amy B.; Reiser, Sally; Simone, Angelo; Katzman, Debra K.; Cafazzo, Joseph A.; Palmert, Mark R. | 2017 | A Mobile App for the Self-Management of Type 1 Diabetes Among Adolescents: A Randomized Controlled Trial |
| Grady, M.; Katz, L. B.; Cameron, H.; Levy, B. L. | 2017 | Diabetes App-Related Text Messages From Health Care Professionals in Conjunction With a New Wireless Glucose Meter With a Color Range Indicator Improves Glycemic Control in Patients With Type 1 and Type 2 Diabetes: Randomized Controlled Trial |
| Greer, J. A.; Jacobs, J.; Pensak, N.; MacDonald, J. J.; Fuh, C. X.; Perez, G. K.; Ward, A.; Tallen, C.; Muzikansky, A.; Traeger, L.; Penedo, F. J.; El-Jawahri, A.; Safren, S. A.; Pirl, W. F.; Temel, J. S. | 2019 | Randomized Trial of a Tailored Cognitive-Behavioral Therapy Mobile Application for Anxiety in Patients with Incurable Cancer |
| Gunawardena, K. C.; Jackson, R.; Robinett, I.; Dhaniska, L.; Jayamanne, S.; Kalpani, S.; Muthukuda, D. | 2019 | The Influence of the Smart Glucose Manager Mobile Application on Diabetes Management |
| Guo, H.; Zhang, Y.; Li, P.; Zhou, P.; Chen, L. M.; Li, S. Y. | 2018 | Evaluating the effects of mobile health intervention on weight management, glycemic control and pregnancy outcomes in patients with gestational diabetes mellitus |
| Gustafson, David H.; McTavish, Fiona M.; Chih, Ming-Yuan; Atwood, Amy K.; Johnson, Roberta A.; Boyle, Michael G.; Levy, Michael S.; Driscoll, Hilary; Chisholm, Steven M.; Dillenburg, Lisa; Isham, Andrew; Shah, Dhavan | 2014 | A smartphone application to support recovery from alcoholism: a randomized clinical trial |
| Hacker, E.; Horsham, C.; Vagenas, D.; Jones, L.; Lowe, J.; Janda, M. | 2018 | A Mobile Technology Intervention With Ultraviolet Radiation Dosimeters and Smartphone Apps for Skin Cancer Prevention in Young Adults: Randomized Controlled Trial |
| Hales, Sarah; Turner-McGrievy, Gabrielle M.; Wilcox, Sara; Fahim, Arjang; Davis, Rachel E.; Huhns, Michael; Valafar, Homayoun | 2016 | Social networks for improving healthy weight loss behaviors for overweight and obese adults: A randomized clinical trial of the social pounds off digitally (Social POD) mobile app |
| Hammonds, Tracy; Rickert, Krista; Goldstein, Carly; Gathright, Emily; Gilmore, Sarah; Derflinger, Bethany; Bennett, Brooke; Sterns, Anthony; Drew, Barbara L.; Hughes, Joel W. | 2015 | Adherence to antidepressant medications: a randomized controlled trial of medication reminding in college students |
| Hannon, T. S.; Yazel-Smith, L. G.; Hatton, A. S.; Stanton, J. L.; Moser, E. A. S.; Li, X.; Carroll, A. E. | 2018 | Advancing diabetes management in adolescents: Comparative effectiveness of mobile self-monitoring blood glucose technology and family-centered goal setting |
| Harries, Tim; Eslambolchilar, Parisa; Rettie, Ruth; Stride, Chris; Walton, Simon; van Woerden, Hugo C. | 2016 | Effectiveness of a smartphone app in increasing physical activity amongst male adults: a randomised controlled trial |
| Hartin, Pj; Nugent, Cd; McClean, Si; Cleland, I; Tschanz, Jat; Clark, Cj; Norton, Mc | 2016 | The empowering role of mobile apps in behavior change interventions: the gray matters randomized controlled trial |
| Hartman, S. J.; Nelson, S. H.; Cadmus-Bertram, L. A.; Patterson, R. E.; Parker, B. A.; Pierce, J. P. | 2016 | Technology- and Phone-Based Weight Loss Intervention: Pilot RCT in Women at Elevated Breast Cancer Risk |
| Hassandra, Mary; Lintunen, Taru; Hagger, Martin S.; Heikkinen, Risto; Vanhala, Mauno; Kettunen, Tarja | 2017 | An mHealth App for Supporting Quitters to Manage Cigarette Cravings With Short Bouts of Physical Activity: A Randomized Pilot Feasibility and Acceptability Study |
| Hides, L; Quinn, C; Cockshaw, W; Stoyanov, S; Zelenko, O; Johnson, D; Tjondronegoro, D; Quek, L-H; Kavanagh, Dj | 2018 | Efficacy and outcomes of a mobile app targeting alcohol use in young people |
| Hides, L.; Dingle, G.; Quinn, C.; Stoyanov, S. R.; Zelenko, O.; Tjondronegoro, D.; Johnson, D.; Cockshaw, W.; Kavanagh, D. J. | 2019 | Efficacy and Outcomes of a Music-Based Emotion Regulation Mobile App in Distressed Young People: Randomized Controlled Trial |
| Hildebrandt, T.; Michaelides, A.; Mackinnon, D.; Greif, R.; DeBar, L.; Sysko, R. | 2017 | Randomized controlled trial comparing smartphone assisted versus traditional guided self-help for adults with binge eating |
| Himelhoch, S.; Kreyenbuhl, J.; Palmer-Bacon, J.; Chu, M.; Brown, C.; Potts, W. | 2016 | Pilot feasibility study of Heart2HAART: a smartphone application to assist with adherence among substance users living with HIV |
| Holmen, Heidi; Torbjornsen, Astrid; Wahl, Astrid Klopstad; Jenum, Anne Karen; Smastuen, Milada Cvancarova; Arsand, Eirik; Ribu, Lis | 2014 | A Mobile Health Intervention for Self-Management and Lifestyle Change for Persons With Type 2 Diabetes, Part 2: One-Year Results From the Norwegian Randomized Controlled Trial RENEWING HEALTH |
| Horsch, Corine Hg; Lancee, Jaap; Griffioen-Both, Fiemke; Spruit, Sandor; Fitrianie, Siska; Neerincx, Mark A.; Beun, Robbert Jan; Brinkman, Willem-Paul | 2017 | Mobile Phone-Delivered Cognitive Behavioral Therapy for Insomnia: A Randomized Waitlist Controlled Trial |
| Hurkmans, E.; Matthys, C.; Bogaerts, A.; Scheys, L.; Devloo, K.; Seghers, J. | 2018 | Face-to-Face Versus Mobile Versus Blended Weight Loss Program: Randomized Clinical Trial.[Erratum appears in JMIR Mhealth Uhealth. 2018 Mar 15;6(3):e10159; PMID: 29543184] |
| Ipjian, Michelle L.; Johnston, Carol S. | 2017 | Smartphone technology facilitates dietary change in healthy adults |
| Irvine, A. Blair; Russell, Holly; Manocchia, Michael; Mino, David E.; Cox Glassen, Terri; Morgan, Rebecca; Gau, Jeff M.; Birney, Amelia J.; Ary, Dennis V. | 2015 | Mobile-Web app to self-manage low back pain: randomized controlled trial |
| Irwin, B; Kurz, D; Chalin, P; Thompson, N | 2016 | Testing the Efficacy of OurSpace, a Brief, Group Dynamics-Based Physical Activity Intervention: A Randomized Controlled Trial |
| Ivanova, Ekaterina; Lindner, Philip; Ly, Kien Hoa; Dahlin, Mats; Vernmark, Kristofer; Andersson, Gerhard; Carlbring, Per | 2016 | Guided and unguided Acceptance and Commitment Therapy for social anxiety disorder and/or panic disorder provided via the Internet and a smartphone application: A randomized controlled trial |
| Järvelä-Reijonen, E.; Karhunen, L.; Sairanen, E.; Muotka, J.; Lindroos, S.; Laitinen, J.; Puttonen, S.; Peuhkuri, K.; Hallikainen, M.; PihlajamÃ¤ki, J.; Korpela, R.; Ermes, M.; Lappalainen, R.; Kolehmainen, M. | 2018 | The effects of acceptance and commitment therapy on eating behavior and diet delivered through face-to-face contact and a mobile app: a randomized controlled trial |
| Jeon, J. H. | 2016 | Evaluation of a smartphone application for self-care performance of patients with chronic hepatitis B: A randomized controlled trial |
| Johnston, Nina; Bodegard, Johan; Jerstrom, Susanna; Akesson, Johanna; Brorsson, Hilja; Alfredsson, Joakim; Albertsson, Per A.; Karlsson, Jan-Erik; Varenhorst, Christoph | 2016 | Effects of interactive patient smartphone support app on drug adherence and lifestyle changes in myocardial infarction patients: A randomized study |
| Kahn, Janet R.; Collinge, William; Soltysik, Robert | 2016 | Post-9/11 Veterans and Their Partners Improve Mental Health Outcomes with a Self-directed Mobile and Web-based Wellness Training Program: A Randomized Controlled Trial |
| Kakoschke, N.; Hawker, C.; Castine, B.; de Courten, B.; Verdejo-Garcia, A. | 2018 | Smartphone-based cognitive bias modification training improves healthy food choice in obesity: A pilot study |
| Karhula, Tuula; Vuorinen, Anna-Leena; Raapysjarvi, Katja; Pakanen, Mira; Itkonen, Pentti; Tepponen, Merja; Junno, Ulla-Maija; Jokinen, Tapio; van Gils, Mark; Lahteenmaki, Jaakko; Kohtamaki, Kari; Saranummi, Niilo | 2015 | Telemonitoring and Mobile Phone-Based Health Coaching Among Finnish Diabetic and Heart Disease Patients: Randomized Controlled Trial |
| Kennard, Betsy D.; Goldstein, Tina; Foxwell, Aleksandra A.; McMakin, Dana L.; Wolfe, Kristin; Biernesser, Candice; Moorehead, Alexandra; Douaihy, Antoine; Zullo, Lucas; Wentroble, Erin; Owen, Victoria; Zelazny, Jamie; Iyengar, Satish; Porta, Giovanna; Brent, David | 2018 | As Safe as Possible (ASAP): A brief app-supported inpatient intervention to prevent postdischarge suicidal behavior in hospitalized, suicidal adolescents |
| Kennelly, M. A.; Ainscough, K.; Lindsay, K. L.; O'Sullivan, E.; Gibney, E. R.; McCarthy, M.; Segurado, R.; DeVito, G.; Maguire, O.; Smith, T.; Hatunic, M.; McAuliffe, F. M. | 2018 | Pregnancy Exercise and Nutrition With Smartphone Application Support: A Randomized Controlled Trial |
| Kim, J. W.; Ryu, B.; Cho, S.; Heo, E.; Kim, Y.; Lee, J.; Jung, S. Y.; Yoo, S. | 2019 | Impact of personal health records and wearables on health outcomes and patient response: Three-arm randomized controlled trial |
| Kirwan, Morwenna; Vandelanotte, Corneel; Fenning, Andrew; Duncan, Mitch J. | 2013 | Diabetes self-management smartphone application for adults with type 1 diabetes: randomized controlled trial |
| Klee, P.; Bussien, C.; Castellsague, M.; Combescure, C.; Dirlewanger, M.; Girardin, C.; Mando, J. L.; Perrenoud, L.; Salomon, C.; Schneider, F.; Schwitzgebel, V. M. | 2018 | An Intervention by a Patient-Designed Do-It-Yourself Mobile Device App Reduces HbA1c in Children and Adolescents with Type 1 Diabetes: A Randomized Double-Crossover Study |
| Kleinman, Nora J.; Shah, Avani; Shah, Sanjiv; Phatak, Sanjeev; Viswanathan, Vijay | 2017 | Improved Medication Adherence and Frequency of Blood Glucose Self-Testing Using an m-Health Platform Versus Usual Care in a Multisite Randomized Clinical Trial Among People with Type 2 Diabetes in India |
| Kosse, R. C.; Bouvy, M. L.; de Vries, T. W.; Koster, E. S. | 2019 | Effect of a mHealth intervention on adherence in adolescents with asthma: A randomized controlled trial |
| Krishnan, N.; Elf, J. L.; Chon, S.; Golub, J. E. | 2018 | COach2Quit: a pilot randomized controlled trial of a personal carbon monoxide monitor for smoking cessation |
| Kuhn, Eric; Kanuri, Nitya; Hoffman, Julia E.; Garvert, Donn W.; Ruzek, Josef I.; Taylor, C. Barr | 2017 | A randomized controlled trial of a smartphone app for posttraumatic stress disorder symptoms |
| Kwon, H.; Lee, S.; Jung, E. J.; Kim, S.; Lee, J. K.; Kim, D. K.; Kim, T. H.; Lee, S. H.; Lee, M. K.; Song, S.; et al. | 2018 | An mhealth management platform for patients with chronic obstructive pulmonary disease (efil breath): randomized controlled trial |
| Laing, B. Y.; Mangione, C. M.; Tseng, C. H.; Leng, M.; Vaisberg, E.; Mahida, M.; Bholat, M.; Glazier, E.; Morisky, D. E.; Bell, D. S. | 2014 | Effectiveness of a smartphone application for weight loss compared with usual care in overweight primary care patients |
| Lakshminarayana, R; Wang, D; Burn, D; Chaudhuri, Kr; Galtrey, C; Guzman, Nv; Hellman, B; James, B; Pal, S; Stamford, J; Steiger, M; Stott, Rw; Teo, J; Barker, Ra; Wang, E; Bloem, Br; Eijk, M; Rochester, L; Williams, A | 2017 | Using a smartphone-based self-management platform to support medication adherence and clinical consultation in Parkinson's disease |
| Lappalainen, P.; Kaipainen, K.; Lappalainen, R.; HoffrÃ©n, H.; MyllymÃ¤ki, T.; Kinnunen, M. L.; Mattila, E.; Happonen, A. P.; Rusko, H.; Korhonen, I. | 2013 | Feasibility of a personal health technology-based psychological intervention for men with stress and mood problems: Randomized controlled pilot trial |
| Lee, B. J.; Park, Y. H.; Lee, J. Y.; Kim, S. J.; Jang, Y.; Lee, J. I. | 2019 | Smartphone Application Versus Pedometer to Promote Physical Activity in Prostate Cancer Patients |
| Lee, J.; Lee, M.; Lim, T.; Kim, T.; Kim, S.; Suh, D.; Lee, S.; Yoon, B. | 2017 | Effectiveness of an application-based neck exercise as a pain management tool for office workers with chronic neck pain and functional disability: A pilot randomized trial |
| Levin, M. E.; Haeger, J.; An, W.; Twohig, M. P. | 2018 | Comparing Cognitive Defusion and Cognitive Restructuring Delivered Through a Mobile App for Individuals High in Self-Criticism |
| Levin, M. E.; Pierce, B.; Schoendorff, B. | 2017 | The acceptance and commitment therapy matrix mobile app: A pilot randomized trial on health behaviors |
| Lin, I. M. | 2018 | Effects of a cardiorespiratory synchronization training mobile application on heart rate variability and electroencephalography in healthy adults |
| Lüdtke, T.; Pult, L. K.; SchrÃ¶der, J.; Moritz, S.; BÃ¼cker, L. | 2018 | A randomized controlled trial on a smartphone self-help application (Be Good to Yourself) to reduce depressive symptoms |
| Ly, Kien Hoa; Topooco, Naira; Cederlund, Hanna; Wallin, Anna; Bergstrom, Jan; Molander, Olof; Carlbring, Per; Andersson, Gerhard | 2015 | Smartphone-Supported versus Full Behavioural Activation for Depression: A Randomised Controlled Trial |
| Mackintosh, Margaret-Anne; Niehaus, James; Taft, Casey T.; Marx, Brian P.; Grubbs, Kathleen; Morland, Leslie A. | 2017 | Using a Mobile Application in the Treatment of Dysregulated Anger Among Veterans |
| Mameli, C.; Brunetti, D.; Colombo, V.; Bedogni, G.; Schneider, L.; Penagini, F.; Borsani, B.; Zuccotti, G. | 2018 | Combined use of a wristband and a smartphone to reduce body weight in obese children: Randomized controlled trial |
| Mangieri, C. W.; Johnson, R. J.; Sweeney, L. B.; Choi, Y. U.; Wood, J. C. | 2019 | Mobile health applications enhance weight loss efficacy following bariatric surgery |
| Mantani, Akio; Kato, Tadashi; Furukawa, Toshi A.; Horikoshi, Masaru; Imai, Hissei; Hiroe, Takahiro; Chino, Bun; Funayama, Tadashi; Yonemoto, Naohiro; Zhou, Qi; Kawanishi, Nao | 2017 | Smartphone Cognitive Behavioral Therapy as an Adjunct to Pharmacotherapy for Refractory Depression: Randomized Controlled Trial |
| Marasinghe, Rohana B.; Edirippulige, Sisira; Kavanagh, David; Smith, Anthony; Jiffry, Mohamad T. M. | 2012 | Effect of mobile phone-based psychotherapy in suicide prevention: A randomized controlled trial in Sri Lanka |
| Oh, Seo Jin; Seo, Sungmin; Lee, Ji Hyun; Song, Myeong Ju; Shin, Min-Sup | 2017 | Effects of smartphone-based memory training for older adults with subjective memory complaints: a randomized controlled trial |
| Marquez Contreras, E.; Marquez Rivero, S.; Rodriguez Garcia, E.; Lopez-Garcia-Ramos, L.; Pastoriza Vilas, J. C.; Baldonedo Suarez, A.; Gracia Diez, C.; Gil Guillen, V.; Martell Claros, N.; Compliance Group of Spanish Society of, Hypertension | 2018 | Specific hypertension smartphone app to improve medication adherence in hypertension: a cluster-randomized trial |
| Martin, Ss; Feldman, Di; Blumenthal, Rs; Jones, Sr; Post, Ws; McKibben, Ra; Michos, Ed; Ndumele, Ce; Ratchford, Ev; Coresh, J; Blaha, Mj | 2015 | mActive: A Randomized Clinical Trial of an Automated mHealth Intervention for Physical Activity Promotion |
| Mayer, Deborah K.; Landucci, Gina; Awoyinka, Lola; Atwood, Amy K.; Carmack, Cindy L.; Demark-Wahnefried, Wendy; McTavish, Fiona; Gustafson, David H. | 2018 | SurvivorCHESS to increase physical activity in colon cancer survivors: Can we get them moving? |
| Mertens, Alexander; Brandl, Christopher; Miron-Shatz, Talya; Schlick, Christopher; Neumann, Till; Kribben, Andreas; Meister, Sven; Diamantidis, Clarissa Jonas; Albrecht, Urs-Vito; Horn, Peter; Becker, Stefan | 2016 | A mobile application improves therapy-adherence rates in elderly patients undergoing rehabilitation: A crossover design study comparing documentation via iPad with paper-based control |
| Mira, Jose Joaquin; Navarro, Isabel; Botella, Federico; Borras, Fernando; Nuno-Solinis, Roberto; Orozco, Domingo; Iglesias-Alonso, Fuencisla; Perez-Perez, Pastora; Lorenzo, Susana; Toro, Nuria | 2014 | A Spanish pillbox app for elderly patients taking multiple medications: randomized controlled trial |
| Miremberg, H.; Ben-Ari, T.; Betzer, T.; Raphaeli, H.; Gasnier, R.; Barda, G.; Bar, J.; Weiner, E. | 2018 | The impact of a daily smartphone-based feedback system among women with gestational diabetes on compliance, glycemic control, satisfaction, and pregnancy outcome: a randomized controlled trial |
| Mollard, E.; Michaud, K. | 2018 | A Mobile App With Optical Imaging for the Self-Management of Hand Rheumatoid Arthritis: Pilot Study |
| Morawski | 2018 | Association of a Smartphone Application With Medication Adherence and Blood Pressure Control: The MedISAFE-BP Randomized Clinical Trial |
| Mummah, S.; Robinson, T. N.; Mathur, M.; Farzinkhou, S.; Sutton, S.; Gardner, C. D. | 2017 | Effect of a mobile app intervention on vegetable consumption in overweight adults: A randomized controlled trial |
| Nezami, B. T.; Ward, D. S.; Lytle, L. A.; Ennett, S. T.; Tate, D. F. | 2017 | A mHealth randomized controlled trial to reduce sugar-sweetened beverage intake in preschool-aged children |
| Ni, Z.; Liu, C.; Wu, B.; Yang, Q.; Douglas, C.; Shaw, R. J. | 2018 | An mHealth intervention to improve medication adherence among patients with coronary heart disease in China: Development of an intervention |
| Nollen, Nicole L.; Mayo, Matthew S.; Carlson, Susan E.; Rapoff, Michael A.; Goggin, Kathy J.; Ellerbeck, Edward F. | 2014 | Mobile technology for obesity prevention: a randomized pilot study in racial- and ethnic-minority girls |
| Nyström, C. D.; Sandin, S.; Henriksson, P.; Henriksson, H.; Trolle-Lagerros, Y.; Larsson, C.; Maddison, R.; Ortega, F. B.; Pomeroy, J.; Ruiz, J. R.; Silfvernagel, K.; Timpka, T.; LÃ¶f, M. | 2017 | Mobile-based intervention intended to stop obesity in preschool-aged children: The MINISTOP randomized controlled trial |
| O'Toole, M. S.; Arendt, M. B.; Pedersen, C. M. | 2019 | Testing an App-Assisted Treatment for Suicide Prevention in a Randomized Controlled Trial: Effects on Suicide Risk and Depression |
| Oh, S. J.; Seo, S.; Lee, J. H.; Song, M. J.; Shin, M. S. | 2018 | Effects of smartphone-based memory training for older adults with subjective memory complaints: a randomized controlled trial |
| Ormel, H. L.; van der Schoot, G. G. F.; Westerink, N. D. L.; Sluiter, W. J.; Gietema, J. A.; Walenkamp, A. M. E. | 2018 | Self-monitoring physical activity with a smartphone application in cancer patients: a randomized feasibility study (SMART-trial) |
| Perera, Anna I.; Thomas, Mark G.; Moore, John O.; Faasse, Kate; Petrie, Keith J. | 2014 | Effect of a smartphone application incorporating personalized health-related imagery on adherence to antiretroviral therapy: a randomized clinical trial |
| Petrella, R. J.; Stuckey, M. I.; Shapiro, S.; Gill, D. P. | 2014 | Mobile health, exercise and metabolic risk: A randomized controlled trial |
| Petrella, Robert J.; Gill, Dawn P.; Zou, Guangyong; De Cruz, Ashleigh; Riggin, Brendan; Bartol, Cassandra; Danylchuk, Karen; Hunt, Kate; Wyke, Sally; Gray, Cindy M.; Bunn, Christopher; Zwarenstein, Merrick | 2017 | Hockey Fans in Training: A Pilot Pragmatic Randomized Controlled Trial |
| Plotnikoff, Ronald C.; Wilczynska, Magdalena; Cohen, Kristen E.; Smith, Jordan J.; Lubans, David R. | 2017 | Integrating smartphone technology, social support and the outdoor physical environment to improve fitness among adults at risk of, or diagnosed with, Type 2 Diabetes: Findings from the 'eCoFit' randomized controlled trial |
| Plow, M.; Golding, M. | 2017 | Using mHealth Technology in a Self-Management Intervention to Promote Physical Activity Among Adults With Chronic Disabling Conditions: Randomized Controlled Trial |
| Quinn, Charlene C.; Shardell, Michelle D.; Terrin, Michael L.; Barr, Erik A.; Ballew, Shoshana H.; Gruber-Baldini, Ann L. | 2011 | Cluster-randomized trial of a mobile phone personalized behavioral intervention for blood glucose control |
| Rabinovich, R. A.; Buttery, S. C.; Puhan, M. A.; Troosters, T.; Janssens, W.; Van den Brande, P.; Demeyer, H.; Spruyt, M.; Loeckx, M.; Hornikx, M.; et al. | 2017 | Physical activity is increased by a 12-week semiautomated telecoaching programme in patients with COPD: a multicentre randomised controlled trial |
| Recio-Rodríguez, J. I.; Rodriguez-Sanchez, E.; Martin-Cantera, C.; Martinez-Vizcaino, V.; Arietaleanizbeaskoa, M. S.; Gonzalez-Viejo, N.; Menendez-Suarez, M.; GÃ³mez-Marcos, M. A.; Garcia-Ortiz, L.; on behalf of the, Evident Investigators group | 2018 | Combined use of a healthy lifestyle smartphone application and usual primary care counseling to improve arterial stiffness, blood pressure and wave reflections: a Randomized Controlled Trial (EVIDENT II Study) |
| Reid, Sophie C.; Kauer, Sylvia D.; Hearps, Stephen J. C.; Crooke, Alexander H. D.; Khor, Angela S.; Sanci, Lena A.; Patton, George C. | 2011 | A mobile phone application for the assessment and management of youth mental health problems in primary care: a randomised controlled trial |
| Roepke, Ann Marie; Jaffee, Sara R.; Riffle, Olivia M.; McGonigal, Jane; Broome, Rose; Maxwell, Bez | 2015 | Randomized controlled trial of SuperBetter, a smartphone-based/internet-based self-help tool to reduce depressive symptoms |
| Rosen, K. D.; Paniagua, S. M.; Kazanis, W.; Jones, S.; Potter, J. S. | 2018 | Quality of life among women diagnosed with breast Cancer: A randomized waitlist controlled trial of commercially available mobile app-delivered mindfulness training |
| Ross, Kathryn M.; Wing, Rena R. | 2016 | Impact of newer self-monitoring technology and brief phone-based intervention on weight loss: A randomized pilot study |
| Santo, K.; Singleton, A.; Rogers, K.; Thiagalingam, A.; Chalmers, J.; Chow, C. K.; Redfern, J. | 2019 | Medication reminder applications to improve adherence in coronary heart disease: A randomised clinical trial |
| Sarfo, F. S.; Treiber, F.; Gebregziabher, M.; Adamu, S.; Nichols, M.; Singh, A.; Obese, V.; Sarfo-Kantanka, O.; Sakyi, A.; Adu-Darko, N.; Tagge, R.; Agyei-Frimpong, M.; Kwarteng, N.; Badu, E.; Mensah, N.; Ampofo, M.; Jenkins, C.; Ovbiagele, B.; on behalf of, Pings Team | 2018 | Phone-based intervention for blood pressure control among Ghanaian stroke survivors: A pilot randomized controlled trial |
| Schatz, Jeffrey; Schlenz, Alyssa M.; McClellan, Catherine B.; Puffer, Eve S.; Hardy, Steven; Pfeiffer, Matthew; Roberts, Carla W. | 2015 | Changes in coping, pain, and activity after cognitive-behavioral training: A randomized clinical trial for pediatric sickle cell disease using smartphones |
| Schlosser, D. A.; Campellone, T. R.; Truong, B.; Etter, K.; Vergani, S.; Komaiko, K.; Vinogradov, S. | 2018 | Efficacy of PRIME, a Mobile App Intervention Designed to Improve Motivation in Young People With Schizophrenia |
| Schnall, R.; Cho, H.; Mangone, A.; Pichon, A.; Jia, H. | 2018 | Mobile Health Technology for Improving Symptom Management in Low Income Persons Living with HIV |
| Shorey, S.; Ng, Y. P. M.; Ng, E. D.; Siew, A. L.; MÃ¶relius, E.; Yoong, J.; Gandhi, M. | 2019 | Effectiveness of a Technology-Based Supportive Educational Parenting Program on Parental Outcomes (Part 1): Randomized Controlled Trial |
| Song, M.; Kanaoka, H. | 2018 | Effectiveness of mobile application for menstrual management of working women in Japan: randomized controlled trial and medical economic evaluation |
| Spring, B.; Pellegrini, C.; McFadden, H. G.; Pfammatter, A. F.; Stump, T. K.; Siddique, J.; King, A. C.; Hedeker, D. | 2018 | Multicomponent mHealth intervention for large, sustained change in multiple diet and activity risk behaviors: The make better choices 2 randomized controlled trial |
| Spring, Bonnie; Pellegrini, Christine A.; Pfammatter, Angela; Duncan, Jennifer M.; Pictor, Alex; McFadden, H. Gene; Siddique, Juned; Hedeker, Donald | 2017 | Effects of an abbreviated obesity intervention supported by mobile technology: The ENGAGED randomized clinical trial |
| Stephens, J. D.; Yager, A. M.; Allen, J. | 2017 | Smartphone Technology and Text Messaging for Weight Loss in Young Adults: a Randomized Controlled Trial |
| Stiles-Shields, C.; Montague, E.; Kwasny, M. J.; Mohr, D. C. | 2018 | Behavioral and cognitive intervention strategies delivered via coached apps for depression: Pilot trial |
| Stukus, D. R.; Farooqui, N.; Strothman, K.; Ryan, K.; Zhao, S.; Stevens, J. H.; Cohen, D. M. | 2018 | Real-world evaluation of a mobile health application in children with asthma |
| Subasinghe, A. K.; Garland, S. M.; Gorelik, A.; Tay, I.; Wark, J. D. | 2019 | Using Mobile Technology to Improve Bone-Related Lifestyle Risk Factors in Young Women With Low Bone Mineral Density: Feasibility Randomized Controlled Trial |
| Sun, C.; Sun, L.; Xi, S.; Zhang, H.; Wang, H.; Feng, Y.; Deng, Y.; Xiao, X.; Wang, G.; Gao, Y. | 2019 | Mobile phoneâ€“based telemedicine practice in older Chinese patients with type 2 diabetes mellitus: Randomized controlled trial |
| Svetkey, Laura P.; Batch, Bryan C.; Lin, Pao-Hwa; Intille, Stephen S.; Corsino, Leonor; Tyson, Crystal C.; Bosworth, Hayden B.; Grambow, Steven C.; Voils, Corrine; Loria, Catherine; Gallis, John A.; Schwager, Jenifer; Bennett, Gary B. | 2015 | Cell phone intervention for you (CITY): A randomized, controlled trial of behavioral weight loss intervention for young adults using mobile technology |
| Tanaka, K.; Sasai, H.; Wakaba, K.; Murakami, S.; Ueda, M.; Yamagata, F.; Sawada, M.; Takekoshi, K. | 2018 | Professional dietary coaching within a group chat using a smartphone application for weight loss: a randomized controlled trial |
| Teng, M. H.; Hou, Y. M.; Chang, S. H.; Cheng, H. J. | 2019 | Home-delivered attention bias modification training via smartphone to improve attention control in sub-clinical generalized anxiety disorder: A randomized, controlled multi-session experiment |
| Tighe, Joseph; Shand, Fiona; Ridani, Rebecca; Mackinnon, Andrew; De La Mata, Nicole; Christensen, Helen | 2017 | Ibobbly mobile health intervention for suicide prevention in Australian Indigenous youth: a pilot randomised controlled trial |
| Turner-McGrievy, G. M.; Wilcox, S.; BouttÃ©, A.; Hutto, B. E.; Singletary, C.; Muth, E. R.; Hoover, A. W. | 2017 | The Dietary Intervention to Enhance Tracking with Mobile Devices (DIET Mobile) Study: A 6-Month Randomized Weight Loss Trial |
| Turner-McGrievy, Gabrielle; Tate, Deborah | 2011 | Tweets, Apps, and Pods: Results of the 6-Month Mobile Pounds Off Digitally (Mobile POD) Randomized Weight-Loss Intervention Among Adults |
| Valle, C. G.; Deal, A. M.; Tate, D. F. | 2017 | Preventing weight gain in African American breast cancer survivors using smart scales and activity trackers: a randomized controlled pilot study |
| van der Weegen, Sanne; Verwey, Renee; Spreeuwenberg, Marieke; Tange, Huibert; van der Weijden, Trudy; de Witte, Luc | 2015 | It's LiFe! Mobile and Web-Based Monitoring and Feedback Tool Embedded in Primary Care Increases Physical Activity: A Cluster Randomized Controlled Trial |
| van Drongelen, Alwin; Boot, Cecile Rl; Hlobil, Hynek; Twisk, Jos Wr; Smid, Tjabe; van der Beek, Allard J. | 2014 | Evaluation of an mHealth intervention aiming to improve health-related behavior and sleep and reduce fatigue among airline pilots |
| Van Horn, L.; Peaceman, A.; Kwasny, M.; Vincent, E.; Fought, A.; Josefson, J.; Spring, B.; Neff, L. M.; Gernhofer, N. | 2018 | Dietary Approaches to Stop Hypertension Diet and Activity to Limit Gestational Weight: Maternal Offspring Metabolics Family Intervention Trial, a Technology Enhanced Randomized Trial |
| Vorrink, Sigrid N. W.; Kort, Helianthe S. M.; Troosters, Thierry; Zanen, Pieter; Lammers, Jan-Willem J. | 2016 | Efficacy of an mHealth intervention to stimulate physical activity in COPD patients after pulmonary rehabilitation |
| Walsh, Jane C.; Corbett, Teresa; Hogan, Michael; Duggan, Jim; McNamara, Abra | 2016 | An mHealth Intervention Using a Smartphone App to Increase Walking Behavior in Young Adults: A Pilot Study |
| Wang, J.; Cai, C.; Padhye, N.; Orlander, P.; Zare, M. | 2018 | A Behavioral Lifestyle Intervention Enhanced With Multiple-Behavior Self-Monitoring Using Mobile and Connected Tools for Underserved Individuals With Type 2 Diabetes and Comorbid Overweight or Obesity: Pilot Comparative Effectiveness Trial |
| Wang, Q. Q.; Zhao, J.; Huo, X. R.; Wu, L.; Yang, L. F.; Li, J. Y.; Wang, J. | 2018 | Effects of a home care mobile app on the outcomes of discharged patients with a stoma: a randomised controlled trial |
| Wayne, Noah; Perez, Daniel F.; Kaplan, David M.; Ritvo, Paul | 2015 | Health coaching reduces HbA1c in type 2 diabetic patients from a lower-socioeconomic status community:A randomized controlled trial |
| Weisman, O.; Schonherz, Y.; Harel, T.; Efron, M.; Elazar, M.; Gothelf, D. | 2018 | Testing the Efficacy of a Smartphone Application in Improving Medication Adherence, Among Children with ADHD |
| Widmer, R. J.; Allison, T. G.; Lennon, R.; Lopez-Jimenez, F.; Lerman, L. O.; Lerman, A. | 2017 | Digital health intervention during cardiac rehabilitation: A randomized controlled trial |
| Yu, Y.; Yan, Q.; Li, H.; Wang, L.; Wang, H.; Zhang, Y.; Xu, L.; Tang, Z.; Yan, X.; Chen, Y.; He, H.; Chen, J.; Feng, B. | 2019 | Effects of mobile phone application combined with or without self-monitoring of blood glucose on glycemic control in patients with diabetes: A randomized controlled trial |
| Zairina, Elida; Abramson, Michael J.; McDonald, Christine F.; Li, Jonathan; Dharmasiri, Thanuja; Stewart, Kay; Walker, Susan P.; Paul, Eldho; George, Johnson | 2016 | Telehealth to improve asthma control in pregnancy: A randomized controlled trial |
| Zhou, Weibin; Chen, Min; Yuan, Jingyun; Sun, Yan | 2016 | Welltang - A smart phone-based diabetes management application - Improves blood glucose control in Chinese people with diabetes |
| Zhu, X.; Zhang, W.; Operario, D.; Zhao, Y.; Shi, A.; Zhang, Z.; Gao, P.; Perez, A.; Wang, J.; Zaller, N.; Yang, C.; Sun, Y.; Zhang, H. | 2019 | Effects of a Mobile Health Intervention to Promote HIV Self-testing with MSM in China: A Randomized Controlled Trial |
